# Diversity and spread of cytoplasmic incompatibility genes among maternally inherited symbionts

**DOI:** 10.1371/journal.pgen.1011856

**Published:** 2025-09-09

**Authors:** Julien Amoros, Marie Buysse, Anna Maria Floriano, Bouziane Moumen, Fabrice Vavre, Didier Bouchon, Olivier Duron

**Affiliations:** 1 MIVEGEC, University of Montpellier, CNRS, IRD, Montpellier, France; 2 LBBE, Université Lyon 1, CNRS, VetAgroSup, Villeurbanne, France; 3 EBI, University of Poitiers, CNRS, Poitiers, France; Penn State University, UNITED STATES OF AMERICA

## Abstract

Cytoplasmic Incompatibility (CI) causes embryonic lethality in arthropods, resulting in a significant reduction in reproductive success. In most cases, this reproductive failure is driven by *Wolbachia* endosymbionts through their *cifA*-*cifB* gene pair, whose products disrupts arthropod DNA replication during embryogenesis. While a *cif* pair has been considered a hallmark of *Wolbachia*, its presence and functional significance in other bacterial lineages remains poorly investigated. Here, we conducted a comprehensive survey of 762 genomes spanning non-*Wolbachia* endosymbionts and their close relatives, revealing that the *cif* pair is far more widespread than previously recognized. We identified *cif* loci in 8.4% of the surveyed genomes, with a striking incidence of 17.4% in facultative symbionts. Beyond *Wolbachia*, *cif* pair occurs across eight bacterial genera spanning α-Proteobacteria, γ-Proteobacteria, Mollicutes, and Bacteroidota. Notably, *cif* pair has been identified in several intracellular pathogens of mammals showing high rate of transovarial transmission in their arthropod hosts, suggesting a potential role of *cif* pair and CI in vector-borne disease dynamics. Structural analyses further reveal that the PD(D/E)-XK nucleases and AAA-ATPase-like motifs are consistently conserved across *cif* pairs in all bacterial taxa. Moreover, *cif* pairs are frequently integrated within diverse mobile genetic elements, from transposons to large intact WO prophages in *Wolbachia* and RAGEs in Rickettsiaceae. Phylogenetic analyses reveal recent and potentially ongoing horizontal transfers of *cif* pair between distantly related bacterial lineages, a process potentially facilitated by mobile genetic elements. Indeed, the PDDEXK2 transposase exhibits a phylogenetic pattern consistent with the co-transmission of *cif* genes, suggesting that it may facilitate horizontal transfers of *cif* across bacterial lineages. Furthermore, the detection of endosymbionts harboring *cif* pair in arthropod groups where *Wolbachia* is scarce, such as ticks, suggests that CI may be more widespread than previously known, with significant implications for arthropod symbiosis, reproductive manipulation, and future biocontrol strategies.

## Author summary

Cytoplasmic incompatibility (CI) is a reproductive strategy used by certain bacteria to promote their spread within arthropod populations. These bacteria, inherited through the mother, cause embryo death in specific mating combinations, giving infected females a reproductive advantage. CI has mostly been linked to *Wolbachia*, a common insect endosymbiont, and its *cifA* and *cifB* genes. In this study, we show that these *cif* genes are much more widespread than previously thoughtThey are present in multiple unrelated bacterial groups, but consistently associated with transovarial transmission in arthropod hosts, including some vector-borne pathogens that also infect vertebrates. Our findings suggest that these bacteria may also use CI-like mechanisms to spread. We further show that *cif* genes are often associated with mobile genetic elements, enabling them to jump between species. This reveals that CI may be a far more common and influential force in arthropod biology than recognized, with important implications for evolution, symbiosis, and future strategies to control disease-carrying arthropods.

## Introduction

Cytoplasmic Incompatibility (CI) is a pervasive selfish genetic mechanism shaping arthropod populations [1–3]. It involves conditional reproductive failure induced by maternally inherited bacteria, which manipulate host reproduction to promote their own transmission. CI typically leads to reproductive incompatibility between infected males and uninfected females, or between individuals infected with different bacterial strains, resulting in embryonic death [1–3]. However, infected females can reproduce successfully with uninfected males or males infected with the same bacterial strain, giving them a reproductive advantage [1–3]. This selective pressure favours the reproduction of infected females (the transmitting sex), promoting the rapid spread of CI symbiont within the population [1–3]. By altering reproductive success and compatibility between maternal lineages, CI reshapes population dynamics and rapidly alters gene flow between populations, affecting the host’s mitochondrial diversity and potentially contributing to reproductive isolation [1,4–6]. In the realm of applied science, CI shows significant potential for public health strategies, particularly for the control of vector-borne diseases as best illustrated by the World Mosquito Program [3,7–9].

The α-proteobacterium *Wolbachia* (order Rickettsiales*,* family Anaplasmataceae) is the most extensively studied CI symbiont [10–14]. This bacterium is widespread in insects but also infects a diverse range of other arthropods, including spiders, mites, millipedes, and woodlice, with an estimated prevalence in over 40% of terrestrial arthropod species [15–17]. Beyond *Wolbachia*, several other maternally inherited bacteria also induce CI in arthropods, although these symbionts are generally considered more incidental. Within the Rickettsiales order, at least another genus of the Anaplasmataceae family, *Mesenet* [18,19], and a genus of the Rickettsiaceae family, *Rickettsia*, contain each a strain able to induce CI in their respective insect hosts [20]. CI symbionts are also found across different bacterial classes, such as the γ-proteobacterium *Rickettsiella* (Legionellales, Coxiellaceae), which induces CI in a spider species [21], the mollicutes *Spiroplasma* (Mycoplasmatales, Spiroplasmataceae), which induces CI in a parasitoid wasp [22], and the bacteroidota *Cardinium* (Cytophagales, Amoebophilaceae), which induces CI in several insect and mite species [23–27]. As CI enhances the spread of maternally inherited symbionts through host populations, this significant selective advantage has likely been a key driver of the emergence of CI in multiple bacterial lineages [1,22,27].

The understanding of molecular mechanisms driving CI has emerged from studies on *Wolbachia* in which CI primarily depends on a two-gene system, *cifA* and *cifB* [28,29]. CI operates through a modification-rescue system whereby the *cifA-cifB* pair or *cifB* gene alone modify the sperm in males, and the *cifA* gene can rescue this modification in a compatible cross [28,29]. Consequently, the presence and activity of these two genes are consistently associated with the induction and rescue of CI. Syntenic *cifA* and *cifB* genes are identified in most *Wolbachia* genomes [30–32], with both genes co-transcribed in a polycistronic transcript [28,33] and sharing a common evolutionary history [30,31,34]. This evidence suggests they form a single *cif* operon, though this remains a subject of debate as there is still little evidence of a shared promoter. Additionally, the presence of a predicted hairpin loop between *cifA* and *cifB* may act as a transcriptional terminator, raising the possibility that the observed transcript could result from background expression rather than a genuine operon [33,35]. The *cif* pair can display polymorphic domain architectures including nuclease, deubiquitinase (DUB, also named ubiquitin-like protease 1 or Ulp1), RNA binding, nuclear localization, and other functional domains [3,28–31,33,36]. The earliest modification to the developing sperm is depletion of a long non-coding RNA that regulates the chromatin organization and genome integrity during the histone-to-protamine transition [36]. Subsequent effects by PD-(D/E)XK-like nuclease domains (also named restriction endonucleases, or REases) cause DNA damage during spermatid development, in conjunction with the alterations to sperm chromatin integrity [36]. This work validates *in vitro* work demonstrating CifB can cleave DNA [37]. DUB domain removes ubiquitin from host proteins, playing a role in the stabilization of CifB [38,39]. Additionally, some CifB proteins feature ankyrin repeat domains, which may mediate protein-protein interactions, allowing *Wolbachia* to interface with host cellular machinery [31]. The diversity of *cif* domain sequences and structures are extensively impacted by repeated independent lateral transfers and recombination involving gene conversion [30,31]. This modular architecture, combined with mobile genetic elements such as WO prophage genes, transposons, and retrotransposons - which are frequently associated with *cif* pairs [29,31,40,41] - likely underpins the emergence of the *cif* pair as a recombination hotspot [30,31], driving its diversification through genomic rearrangement or potentially through transduction [42,43]. This diversity provides the *cif* genes with high functional flexibility, potentially enabling *Wolbachia* to adapt its reproductive manipulation strategies to various host species and reproductive contexts [30,31,44].

While CI non-*Wolbachia* symbionts induce similar reproductive phenotypes in their respective hosts, the molecular mechanisms underlying CI in these symbionts remain elusive. Indeed, the *cif* genes are absent in the *Cardinium* and *Spiroplasma* genomes of CI strains, indicating that these genes are not involved in the expression of CI in these bacteria [22,27,45]. However, the discovery of *Mesenet*, a sister genus of *Wolbachia*, whose members also carry *cifA* and *cifB* genes and induce CI in the coconut beetle *Brontispa longissima* [18,19], revealed that other symbionts can effectively mimic *Wolbachia* in terms of reproductive manipulation. This pattern was confirmed in 2024 with the discovery of a *Rickettsia* strain carrying *cif* genes, analogous to those primarily found in *Wolbachia* genomes, which can also induce strong CI in the mirid bug *Nesidiocoris tenuis* [20]. Recent publications reporting the presence of *cif* genes in some other *Rickettsia* genomes, but also in other maternally inherited symbionts and in few insect genomes, further suggest that CI possibly arose in these lineages through horizontal transfers of the *cif* pair accross phylogenetic barriers [30,31,34,44]. Phylogenetic analyses of the *cif* pair showed significant genetic diversity, classified into ten phylogenetic clades (types I-X, [29–31,33]). Types I to IV are primarily associated with *Wolbachia* genomes and have been the focus of extensive investigation in recent years [28–30,36,39,46,47]. In contrast, types V to X exhibit important domain polymorphism and are found in *Wolbachia* and other maternally inherited symbionts [30,31,34]. However, the study of the non-*Wolbachia cif* genes remains largely overlooked, with much of their functional diversity, association with mobile genetic elements in their flanking genomic environment, and evolutionary history still to be understood.

In this study, an extensive analysis of non-*Wolbachia cif* pairs was carried out by examining a broad genomic dataset of maternally inherited symbionts and their close relatives, all distinct from the *Wolbachia* genus. The dataset included publicly available genomes as well as newly sequenced *Rickettsia* genomes. Leveraging this comprehensive dataset, we systematically assessed the distribution, diversity, and domain architecture of *cifA* and *cifB* genes across the genomes of these symbionts and conducted comparative analyses using a representative set of *Wolbachia* genomes as references. We specifically analyzed *cifB*-associated domain sequences and assessed the potential of non-*Wolbachia* symbionts to induce CI. Furthermore, we investigated the genomic regions flanking the *cif* loci, identifying their close association with a diverse array of mobile genetic elements.

## Results

### Distribution of *cif* genes in non-*Wolbachia* symbionts

The genomic analysis of representative non-*Wolbachia* maternally inherited symbionts and their close relatives revealed a scattered distribution of both *cifA* and *cifB* genes across bacterial phylogeny. Among the 754 public genomes and the eight new *Rickettsia* genomes assembled in this study (Table 1), *cif* genes were identified in 64 (8.40%) of them (S1 Table). Within the *cif*-positive genomes, both *cifA* and *cifB* were co-detected in 60 genomes (93.75%), predominantly organized in an pair structure. In contrast, four genomes (6.25%) contain a *cifB* gene copy without any evidence of *cifA*. The number of *cif* pair copies per *cif*-positive genome ranges from one (54/60, 90%) to two (5/60, 8.33%), except for *Rickettsia hoogstraalii* strain RCCE3, which genome carried five pair copies. However, as not all genomes are complete, these copy numbers should be interpreted with caution, as some duplications may have been under-counted.

**Table 1:** Summary metrics of the eight newly assembled *Rickettsia* genomes.

Strain	Genome Size (bp)	GC content (%)	Completeness	Redundancy	N Contigs	Contig N50	Contig L50	Estimated N CDS	Host tick species
*Rickettsia lusitaniae* strain R-Oe	1,576,203	32.273	0.942	1.000	153	15,153	29	1,706	*Ornithodoros erraticus*
*Rickettsia vini* strain IarboMS4	1,314,191	32.407	0.933	1.010	25	120,039	4	1,501	*Ixodes arboricola*
*Rickettsia* sp. strain AdisF19	1,570,282	31.784	0.9333	1.000	84	38,164	15	1,625	*Amblyomma dissimile*
*Rickettsia* sp. strain AdisM1	1,519,432	31.679	0.9333	1.000	76	38,276	14	1,567	*Amblyomma dissimile*
*Rickettsia* sp. strain AdisP2	1,503,972	31.61	0.933	1.010	1	1,503,972	1	1,534	*Amblyomma dissimile*
*Rickettsia* sp. strain AdisP3Sn	1,467,430	31.573	0.933	1.000	75	38,276	14	1,508	*Amblyomma dissimile*
*Rickettsia raoultii* strain Dreti_100P	1,386,961	32.5	0.943	1.010	84	60,986	6	1,529	*Dermacentor reticulatus*
*Rickettsia raoultii* strain Dreti_100F	1,365,498	32.462	0.943	1.010	76	60,981	6	1,509	*Dermacentor reticulatus*

**Fig 1:**
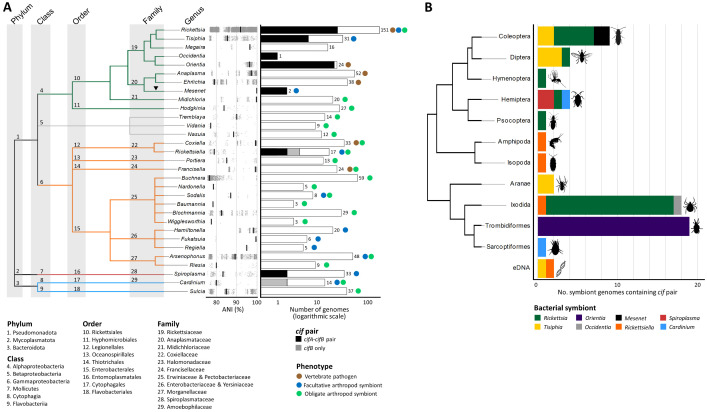
Distribution of *cif* pairs in maternally inherited bacterial symbionts of arthropods and their close relatives, excluding members of the *Wolbachia* genus. (A) Number of genomes containing a *cif* pair across the different genera analyzed, shown on a logarithmic scale. A cladogram depicting the phylogenetic relationships between bacterial genera is shown on the left. The genus *Wolbachia* is not represented here, but its phylogenetic position (▼) as a sister group to *Mesenet* within the Anaplasmataceae family is indicated. The Average Nucleotide identity (ANI) distribution and median are provided for each genus. The total number of genomes analyzed and the phenotype(s) associated are indicated for each genus. (B) Number of symbiont genomes containing at least one *cifA-cifB* pair or *cifB* gene, categorized by the arthropod order from which they were isolated. A cladogram illustrating the phylogenetic relationships of arthropod orders is shown on the left. Open-source images available are used under permissive licenses from Openclipart (https://openclipart.org/share) and Pexels (https://www.pexels.com/license/).

The distribution of *cif* genes shows significant variation across the examined genomes, reflecting both the nature of interactions with arthropod hosts, the phylogenetic constraints, and associated hosts. Specifically, the analysis encompasses genomes of (i) obligate symbionts (n = 276), which are essential for the survival of their arthropod hosts, (ii) facultative symbionts (n = 196), which are not critical for arthropod survival, (iii) their relatives that cause diseases in vertebrates and are transmitted by arthropod vectors (n = 217), and (iv) their relatives of other or unknown phenotypes (n = 73) (S1 Table). In this context, *cif* genes are significantly more prevalent in facultative symbionts (34 out of 196 genomes, 17.35%) compared to obligate symbionts (0/276, 0%; Fisher’s exact tests, *p* < 10^-12^) and vector-borne bacteria causing diseases in vertebrates (21/217, 9.68%; *p* = 0.03) (Fig 1A). In the vector-borne bacterial group, the presence of *cif* genes is limited to the scrub typhus agent *Orientia tsutsugamushi* (initially identified in [41,44]), associated with Trombidiformes mites as known vectors. Indeed, no *cif* genes has been identified in other examined pathogenic species associated with arthropods, such as *R. prowazekii*, the causative agent of epidemic typhus transmitted by lice, or *R. rickettsii* and *R. conorii*, responsible for Rocky Mountain and Mediterranean spotted fevers, respectively, both transmitted by ticks. However, unlike *R. prowazekii*, *R. rickettsii*, and *R. conorii*, which rely predominantly on horizontal transmission, *O. tsutsugamushi* is also maintained in arthropod populations through transovarial transmission, with rates close to 100%, ensuring its persistence across generations [48]. Interestingly, *cif* genes exhibit a similar prevalence in facultative symbionts and bacteria with other or unknown phenotypes (9/73, 12.33%; *p* = 0.36). Notably, *cif* genes are found in bacteria with other or unknown phenotypes (categorized in S1 Table under the ‘other phenotypes’ group), such as *R. heilongjiangensis*, which causes opportunistic human infections following tick bites [49–52] but is also commonly found in unfed tick larvae, suggesting efficient maternal inheritance [53]. Additionally, other intracellular bacteria carrying *cif* genes, such as *R. tamurae, R. argasii,* and *R. hoogstraalii* (also listed S1 Table under the ‘other phenotypes’ group), are documented (or strongly suspected) as opportunistic human pathogens [54–56], although it remains unclear whether they are maternally transmitted in their tick hosts. Nevertheless, *R. hoogstraalii* was initially isolated from embryos of the tick *Ornithodoros capensis* [54] and has since been found in the larvae of several tick species [57,58], a pattern also observed for *R. tamurae* [59], suggesting their maternal transmission. Overall, this pattern supports a specific association between *cif* genes and maternal (transovarial) transmission in arthropods, as *cif* genes are absent from bacteria that infect arthropods but rely predominantly on horizontal (infectious) transmission.

The genomes of representative non-*Wolbachia* maternally inherited symbionts and their close relatives, as examined here, span 31 genera across three bacterial phyla, six classes, and at least nine orders and 11 families (as some β-proteobacteria are not yet assigned to specific orders and families; Fig 1A and S1 Table). However, *cif* genes are more prevalent within the α-proteobacteria class (56 out of 362 genomes, 15.47%) compared to other bacterial class (β-proteobacteria: 0/35, 0%, γ-proteobacteria: 4/281, 1.42%, Flavobacteriia: 0/37, 0%; Fisher’s exact tests, all *p* < 0.008) except for Mollicutes (2/33, 6.06%; *p* = 0.20) and Cytophagia (2/14, 14.28%; *p* = 0.99). Specifically, in the α-proteobacteria, evidence of *cif* genes was commonly found in five genera within the Rickettsiales order: *Rickettsia* (25/151 genomes), *Orientia* (21/24), *Tisiphia* (7/31), *Mesenet* (2/2), and *Occidentia* (1/1). They were also detected in other bacterial taxa, including γ-proteobacteria with Legionellales (genus *Rickettsiella*: 4/17), Mollicutes with Mycoplasmatales (*Spiroplasma*: 2/33), and Cytophagia with Cytophagales (*Cardinium*: 2/14). No *cif* genes were identified in other bacterial genera harboring maternally inherited symbionts (Fig 1A and S1 Table), nor were they detected in *Anaplasma* (0/52) and *Ehrlichia* (0/38), which are disease-causing agents transmitted by ticks, despite their close phylogenetic relationship to *Wolbachia* in the Anaplasmataceae family. Since *Anaplasma* and *Ehrlichia* bacteria are not maternally inherited in ticks, the absence of *cif* genes in these bacteria is consistent with the observed absence of these genes in other arthropod-associated bacteria that rely exclusively on horizontal transmission. Analysis of Average Nucleotide Identity (ANI) further indicates that intragenic genetic diversity does not influence the presence or prevalence of *cif* genes within bacterial genera (GLM binomial and LM tests, p = 0.910 and 0.49, respectively, Fig 1A). For instance, bacteria with high intrageneric genetic diversity, such as *Rickettsiella* (ANI mean ± SE: 81.31 ± 0.44; 75 pairwise comparisons) and *Ehrlichia* (85.46 ± 0.19; 820 pairwise comparisons), can display contrasting *cif* gene patterns, being present in 24% of *Rickettsiella* genomes but entirely absent from *Ehrlichia*.

Regarding host taxonomy, non-*Wolbachia cif* genes were commonly found in bacteria associated with Trombiniformes mites (with *Orientia*), ticks (associated with *Rickettsia*, *Occidentia*, and *Rickettsiella*), and Coleoptera (with *Rickettsia*, *Tisiphia*, and *Mesenet*) (Fig 1B). Notably, ticks exhibited an important diversity of *Rickettsia* species and strains carrying *cif* genes (16/96 genomes; S1 Table). To a lesser extent, *cif* genes were identified in non-*Wolbachia* symbionts hosted by other arachnids (Araneae, Sarcoptiformes), insects (Diptera, Hemiptera, Hymenoptera, Psocodea), as well as crustaceans (Amphipoda, Isopoda), and in metagenome-assembled sequences derived from environmental DNA (eDNA), potentially originating from remnants of arthropods.

### Polymorphism dynamics of toxins and other domains in *cifA* and *cifB*

**Fig 2:**
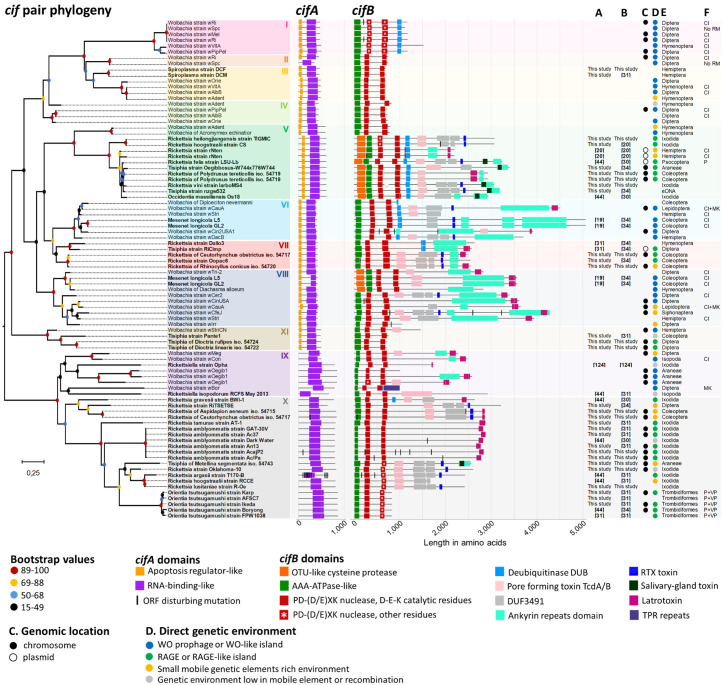
Phylogeny and protein domain architecture of *cifA* and *cifB.* A unique ML phylogeny of the *cif* pair was created by concatenating 164 amino acids from *cifA* and *cifB* (CPREV+G4 substitution model, midpoint-rooted tree, bootstrap values estimated from 1,000 replicates). Non-*Wolbachia* symbiont genera are highlighted in bold. *cif* pair type (I-X) follows the nomenclature established by Tan et al. [31]. The total length of each gene is scaled in amino acids. The domain compositions of the *cifA* and *cifB* genes were colored relative to those described in Martinez et al. [30]. ORF disturbing mutations have been indicated by black vertical lines. First report of domain annotations (A) and phylogenetic analyses (B) of *cif* genes found outside of the *Wolbachia* genus. (C) Genomic location of the *cif* pair in bacteria. Absence of circles indicates that the *cif* pair is located on an unlocalized contig. (D) Direct genetic environment type neighbouring the *cif* pair. (E) Arthropod host order. (F) Known phenotypes induced by the bacteria in their hosts (CI: Cytoplasmic Incompatibility, P: Parthenogenesis, MK: Male killing, No RM: No reproductive manipulation, VP: Vertebrate pathogen).

Both *cifA* and *cifB* genes showed important variation in amino acid sequence length, nature and number of their structural domains (Figs 2 and S1). Phylogenetic analysis, encompassing all non-*Wolbachia cif*-positive symbionts alongside representative *Wolbachia* strains, identified 11 distinct clades of *cif* genes. Ten of these clades align with the previously characterized I-to-X *cif* types described [30,31,34], but we also detected a novel robust clade, which we provisionally designated as type XI. Most *cif* types formed monophyletic clades, with the exception of *cif* type IV, which appeared paraphyletic in our analysis. Additionally, the subclade containing *R. gravesii* and other three *Rickettsia* undetermined species, which was primarily associated with type IX [31], could instead be reassigned to type X. A significant correlation was found across *cif* types between total pair length and the number of predicted domains, with a plateau for values higher than *ca.* 4,000 amino acids with eight or more domains (Polynomial regression analysis based on LOESS values, Adjusted R^2^ = 0.98, *p* < 0.0001, S2 Fig). Furthermore, both the number and composition of domains were significantly associated with *cif* type (Kruskal-Wallis, p < 0.0001, PERMANOVA, *p* < 0.0001, respectively).

*Wolbachia* displayed the greatest diversity of *cif* types, encompassing eight of the 11 types. *Rickettsia* and *Tisiphia* harbored four and three types, respectively. *Rickettsiella cif* genes were restricted to type IX, alongside a small number of *Wolbachia cif* genes, while all *Orientia cif* genes were confined to a subclade within type X, which otherwise included *cif* genes from *Rickettsia* and *Tisiphia*. *Spiroplasma cif* genes grouped with *Wolbachia cif* type III. The two orphan *cifB* genes (*i.e.*, *cifB* without associated *cifA*) from *Cardinium* cluster into types VI and X (S1 Fig).

The *cifA* gene copies have an average length of 595 amino acids (from 359 to 867) and universally contain RNA-binding-like domain (Fig 2). An additional domain, the apoptosis regulator-like domain, is only present in *cifA* types I–VIII and XI, suggesting it was acquired secondarily by the common ancestral *cifA* sequences of these types. In contrast, the *cifB* gene copies exhibit greater polymorphism, both in sequence length (ranging from 696 to 5,065 amino acids, with an average of 2,073 amino acids) and in protein domain composition. The two conserved PD-(D/E)XK nuclease domains are stably maintained across the *cifB* phylogeny, as their presence is required to count as a true hit, and are associated with an AAA-ATPase-like domain, consistent with observations in *Wolbachia* genomes [30] and, more broadly, in bacteria [60]. However, *cifB* types V–VIII exhibit significantly greater domain polymorphism than other types (Kruskal-Wallis test and Dunn’s post hoc tests for multiple comparisons, with FDR correction, *p* < 0.0001). In types I and V–X, protein domain arrangements are predominantly localized to the C-terminal region, with the exception of the N-terminal OTU-like cysteine protease. Frequently observed *cifB* domains included pore-forming toxin TcdA/B (36/111), latrotoxin (33/111), ankyrin repeats (32/111), DUF3491 (28/111), deubiquitinase DUB (27/111), and RTX toxin (22/111) (Fig 2 and S2 Table). Ankyrin repeat domains, often exceeding 2,000 amino acids, were commonly associated with other toxin modules such as TcdA/TcdB, salivary gland toxins, RTX, and latrotoxins. None of the *cif* domains were exclusive to *Wolbachia cifA* or *cifB*; for instance, the DUB domain was also present in *Mesenet*, *Rickettsia*, and *Tisiphia* (Fig 2 and S3 Table). Overall, domain abundance did not differ significantly between Wolbachia and other bacteria (Fisher’s exact tests, all p > 0.05), except for DUF3491, which was more common in Rickettsia pairs (p = 0.02).

Phylogenetic analysis of *cifB* indicated multiple independent gains and losses of C-terminal domains, even within the same *cifB* type (Figs 2 and S1). Separate phylogenies for each predicted domain showed that most sequences clustered by cif type (S3 Fig), suggesting shared evolutionary trajectories. However, several domains showed phylogenetic incongruences, as best exemplified by the latrotoxin domain, which diverges in *cif* type VI and VIII, and the TcdA/TcdB, which varied across types V, VI, VIII, IX, and X (S3 Fig). These patterns support repeated intra-gene recombination events between distantly related *cifB* genes, as previously shown in other studies [30,31,46].

### Predicted functionality of the *cif* pair in CI induction

Among the 107 pairs analysed, 85 contained intact *cifA* and *cifB* coding sequences without internal mutations that could disrupt their functionality. These complete coding sequences were present in *Wolbachia* (31/38), *Orientia* (19/21), *Rickettsia* (19/32), *Tisiphia* (7/7), *Mesenet* (4/4), *Rickettsiella* (2/2), *Spiroplasma* (2/2), and *Occidentia* (1/1) (Fig 2). Conversely, mutations introducing internal stop codons are present in the remaining 22 pairs, primarily in *Wolbachia*, *Rickettsia,* and *Orientia* (Figs 2 and S1 and S2 Table). Of these, seven pairs exhibit mutations in both *cifA* and *cifB*, while 15 show mutations exclusively in *cifB* (Fig 2). Although these mutations could potentially disrupt *cif* functionality, they were observed in the *Rickettsia* rNten strain, which still induces complete CI [20]. As these mutations are not located within the *cif* domains, their ability to induce CI may still be preserved. Additionally, truncated *cifB* genes were identified in *Rickettsia* (4/32), where *cifB* sequences completely lack the C-terminal PD-(D/E)XK nuclease (S1 Fig and S2 Table). This likely leads to the production of an incomplete non-functional protein with the loss of a part of the catalytic site.

Further analysis of *cifB* amino acid sequences highlights the potential of non-*Wolbachia* symbionts to induce CI via the DNase activity of the PD-(D/E)XK nuclease domains. This DNase activity was initially attributed to the presence of three critical catalytic residues (D–E–K) present in both the N- and C-terminal domains [2,37]. Our findings confirm the high conservation of these catalytic residues within the N-terminal PD-(D/E)XK nuclease domains across types II-X (S4 Fig). In the C-terminal domains, these residues are largely preserved across types, except in some type X *cif* pairs from *Rickettsia*, *Tisiphia*, and *Orientia*, where lysine (K) is substituted with glycine (G) or alanine (A) (Figs 2 and S4 and S2 Table). Notably, these preserved N-and C-termini catalytic residues were identified in *cifB* sequences of *Rickettsia* (21/32), *Tisiphia* (6/7), *Mesenet* (4/4), *Rickettsiella* (4/4), *Spiroplasma* (2/2), and *Occidentia* (1/1), alongside those of *Wolbachia* (Fig 2). However, recent studies challenge the requirement of the D–E–K residues for DNase activity, as CI toxicity has been observed even in their absence in both *cif* type I and type X [36,61]. This suggests that the PD-(D/E)XK nuclease domain itself, regardless of the presence of canonical D–E–K residues, may be sufficient to confer CI-inducing DNase activity across all the *cif* types.

### Genomic environments of *cifA-cifB* pairs

**Fig 3:**
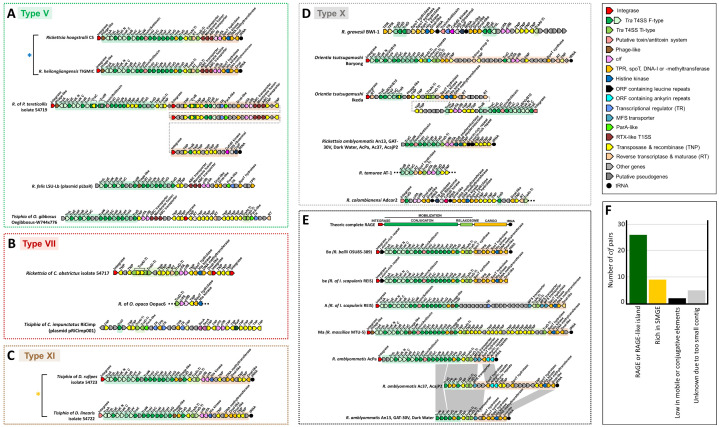
*Rickettsia* Amplified Genetic Elements (RAGE) as recurrent associated environments of *cif* pairs in the Rickettsiaceae family. Gene lengths are not to scale. (A-D) Subsets of RAGE or RAGE-like syntenic environments directly associated with *cif* pairs from: (A) Type V, (B) Type VII, (C) type XI, and (D) Type X. Asterisks highlight homologous *cif* syntenic environments, transferred via lateral gene transfer (LGT) events (blue asterisk) or inherited through cladogenesis (yellow asterisk). Gray dashed lines represent the continuation of the genome onto the next line for visualization purposes. Black dashed lines placed upstream or downstream of certain RAGE or RAGE-like *cif* environments indicate the ends of contigs. For *Rickettsia* of *Polydrusus tereticollis* isolate 54719, diamonds indicate a multi-gene insertion within the RAGE. (E) General schema of a theoretical complete RAGE, modified from Gillespie et al. [71] and Giengkam et al. [66]. The syntenic gene organization of three referenced RAGEs—“Bo” from *R. bellii* OSU-85-389, “A” from *Rickettsia* sp. REIS, and “Ma” from *R. massiliae* MTU-5—are illustrated as examples. We also reference a new RAGE identified in *R. amblyommatis*, which shows variable completeness and gene composition between strains, illustrating the dynamics of RAGE genomic rearrangements. (F) Distribution of *cif* pair direct environments within the Rickettsiaceae family.

**Fig 4:**
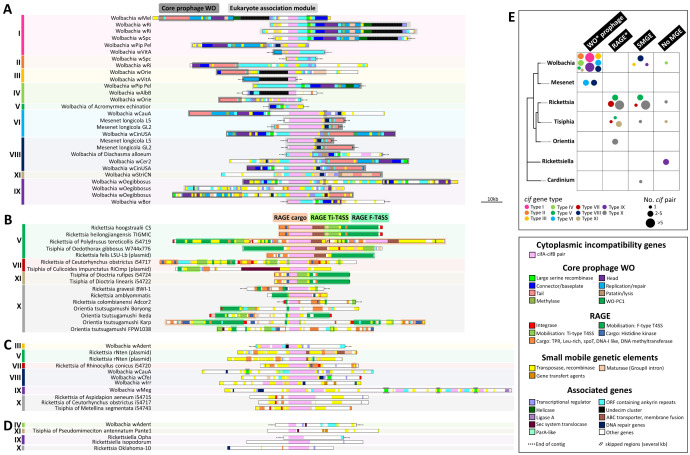
Comparison and genomic location of *cif* pair environments in bacterial symbiont genera. (A-D) Comparison of the *cif* pair environments of *Wolbachia*, *Mesenet*, *Rickettsia*, *Tisiphia*, *Orientia* and *Rickettsiella*. All the environments are aligned on the *cif* pairs oriented in the following order: *cifA*-*cifB*, and ranging according to the *cif* types. The fragment lengths are relative to the indicated scale. Black dashed lines placed upstream or downstream of certain fragments indicate the ends of contigs. (A) WO prophages or WO-like islands environments. The core prophage WO genes and the Eukaryotic association modules are represented in the background following the classification of Bordenstein and Bordenstein [41]. (B) RAGE or RAGE-like environments. The representative F-type T4SS conjugation, Ti-type T4SS relaxation and cargo modules are highlighted in the background with respective colors. (C) Environment enriched in Small Mobile Genetic Elements (SMGE) without WO prophages or RAGE. (D) Environment depleted or devoid of mobile or conjugative elements. (E) Summary of the number of *cif* pairs identified based on bacterial genera, direct genetic environment, and *cif* types. For WO prophage and RAGE, WO-like islands and RAGE-like islands have been combined, respectively (*). SMGE refers to small mobile genetic elements in a restricted environment without WO or RAGE. ‘No MGE’ indicates the absence or insignificance of mobile genetic elements.

A representative subset of 95 *cif* pairs was examined for their genomic location, revealing that 36 are located on main bacterial chromosomes (for *Wolbachia*, *Rickettsia*, *Tisiphia*, and *Orientia*), four on plasmids (*Rickettsia* and *Tisiphia*), and 55 on unlocalized fragments ranging from 3,614–384,641 bp (Fig 2B). In 78 cases, the *cif* genes were found on fragments that were either sufficiently large (≥12,800 bp) or positioned near the center of the fragment, allowing their genomic flanking context to be categorized. The genetic composition of *cif* flanking regions is correlated with both *cif* types and bacterial genera (χ^2^_30_ = 70.82, χ^2^_15_ = 102.68, all *p* ≤ 0.0002, Figs 2C, 3 and 4). Notably, nine out of the ten *cif* type V pairs in *Rickettsia* and *Tisiphia* are located upstream of an RTX-like T1SS, which comprises two ABC transporters and a membrane fusion protein, a genetic feature absent in almost all other *cif* types (Figs 3A and S5). While *cif* types is associated with the composition of flanking regions, the bacterial genus appears to have a stronger influence, as indicated by higher Cramér’s V values (0.67 *vs.* 0.56). This suggests that pre-existing genomic constraints tied to bacterial genera may play a key role in determining the organization of *cif* flanking regions, as exemplified by *Wolbachia* and Rickettsiaceae. Indeed, the majority of *Wolbachia cif* pairs examined (26/32) are located either within or in close association with WO prophages or WO-like genomic islands (Fig 4A and 4E), as previously documented [29,31,32,41]. Notably, *Wolbachia cif* pairs are inserted in eukaryotic association module (EAM), a specialized region of WO prophages strongly linked to, but evolutionarily independent from, the WO core structural genes (*e.g.*, head, tail, replication, repair, connector, baseplate) [41,42]. Similar pattern were observed for the *Mesenet cif* pairs (4/4), which are also associated with WO prophages.

Conversely, we found no evidence of prophage WO associated with *cif* pairs for the other non-*Wolbachia* genomes (except *Mesenet*). Instead, distinct types of mobile genetic elements were detected in association with *cif* pairs in members of the Rickettsiaceae family, specifically in *Rickettsia* (27/28 *cif*-positive genomes), *Tisiphia* (5/6), and *Orientia* (4/5), as well as in the Amoebophilaceae family with the *Cardinium* genus (1/1) (Figs 3A–D and 4B–E). In *Occidentia* and *Spiroplasma*, the physical association between *cif* pairs and mobile genetic elements could not be determined, as these elements are located on unlocalized short contigs. However, in *cif*-positive genomes of *Rickettsia*, *Tisiphia*, and *Orientia*, these mobile genetic elements frequently correspond to conjugative elements known as *Rickettsia* Amplified Genetic Elements (RAGE, Figs 3A–D and 4B), previously identified in some Rickettsiaceae genomes [62–66]. RAGE are primarily organized into two main components: (i) The mobilization module, which includes F- and Ti-type Type IV Secretion System (T4SS) genes (*Tra* genes), and (ii) The cargo module, responsible for transferring closely linked genes within or between genomes through recombination and genomic rearrangements (Fig 3E) [66].

RAGE and *cif* pairs were commonly associated in Rickettsiaceae genomes. Among the 38 *cif* pairs of Rickettsiaceae with large flanking regions available, 26 (68.4%, spanning *cif* types V, VII, XI, and X) were adjacent to RAGE or RAGE-like islands (Figs 3 and 4B). Of these 26 *cif* pairs, 16 (61.5%) were located within complete RAGE elements, which include F-T4SS mobilization genes and cargo-associated genes (*e.g.*, TPR, *SpoT*, and leucine-rich ORFs). Notably, eight *cif* pairs were embedded within the cargo block, positioned between the relaxosome Ti-T4SS genes (*TraA1* and *TraD* Ti-like) and cargo genes (*e.g.*, *R. hoogstraalii* CS, *R. heilongjiangensis* TIGMIC, *Tisiphia* isolate 54722). The sizes of complete RAGE elements varied substantially, ranging from 26,082 bp (*R. amblyommatis* strains) to 184,626 bp (*Rickettsia* isolate 54719), with the largest size due to the duplication of a 48,579 bp RAGE block containing a *cif* pair (S6 Fig). Furthermore, ten *cif* pairs (38.5%) of Rickettsiaceae were located in RAGE-like islands, which were interspersed with *Tra* genes and additional small mobile genetic elements (SMGE). In *Orientia*, the genomic context of *cif* pairs varied by strain. They may be found within RAGE (*e.g.*, strains Boryong and Ikeda), within RAGE-like islands (*e.g.*, strain FPW1038), or flanked by two or three RAGE elements without being part of the cargo (*e.g.*, strain Karp) (Figs 3, 4 and S5).

We also identified SMGE closed to *cif* pairs, such as transposases, recombinases, integrases or retrotransposases (*e.g.*, maturases involved in the processing of group II intron-like retroelements). In some cases, SMGE were adjacent to *cif* pairs inside RAGE (2/11, 18%) and WO prophages (20/27, 74%) (Fig 4A and 4B). We identified an extreme case of duplication of a full transposase-covered fragment flanked by two identical integrases within the same RAGE in *Rickettsia* of *P. tereticollis* isolate 54719. (S6 Fig). In other cases, SMGEs were the only mobile genetic elements associated with *cif* pairs, as observed in both Rickettsiaceae (10/39), *Wolbachia* (5/32), and *Cardinium* (1/1), where no traces of RAGE or WO prophage genes were detected in the vicinity of the *cif* pairs (*e.g.*, *Wolbachia* wIrr, *Rickettsia* rNten, *Tisiphia* isolate 54743; Figs 4C, 4E, S5 and S6).

In rare instances (6/78), *cif* pairs were located in genomic environments that appear to be devoid of mobile genetic elements, as observed in *Rickettsia oklahomensis* strain Oklahoma-10, *Tisiphia* strain Pante 1, *Rickettsiella isopodorum*, *Rickettsiella* sp. strain Opha and *Rickettsiella* sp. strain ETS170220_bin_92 (Fig 4D and 4E). However, for *R. oklahomensis* strain Oklahoma-10 and *Tisiphia* strain Pante 1, relics of RAGE were detected elsewhere in their genomes, suggesting potential genomic rearrangements that may have relocated the *cif* pairs away from the original RAGE context.

### Lateral transfers and recombination of *cif* pairs across symbionts

Phylogenetic reconstructions revealed that distantly related bacteria can harbor the same *cif* types, while bacteria of the same genus may possess distantly related pairs (Fig 2). Notably, although nearly all non-*Wolbachia cif* pairs clustered within types V-to-X, two *Spiroplasma cif* pairs grouped with *Wolbachia* cif type III. Additionally, as *Wolbachia cif* pairs belong to eight types, some clustered with *cif* pairs of distantly related symbionts, as exemplified by *Rickettsia* and *Tisiphia* in types V and XI. While *Rickettsiella cif* pairs were restricted to type IX, they cluster with some *Wolbachia cif* pairs. Type X also includes *Rickettsia*, *Tisiphia,* and *Orientia cif* pairs.

**Fig 5:**
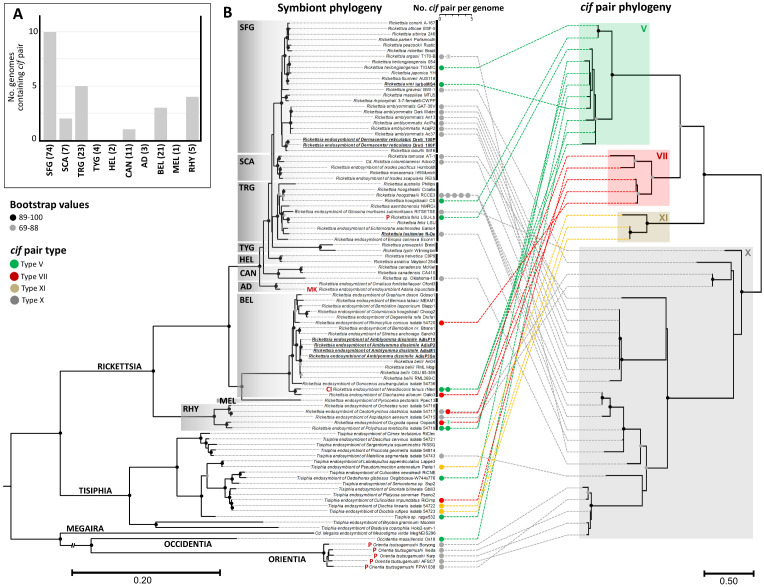
Diversity of the *cif* pair in the Rickettsiaceae family. (A) Number of genomes containing *cif* pairs in the *Rickettsia* genus. *Rickettsia* group names follow the nomenclature established by Davison et al. [72]: SFG, Spotted Fever Group; SCA, Scapularis; TRG, Transitional Group; TYG, Typhus Group; HEL, Helvetica; CAN, Canadensis; AD, Adalia; BEL, Belli; MEL, Meloidae; RHY, Rhyzobius. The total number of genomes analyzed for each *Rickettsia* group is indicated in parentheses. (B) Comparison of the whole-genome and *cif* pair phylogenies within the Rickettsiaceae family. The corresponding whole-genome and *cif* pair phylogenies for Rickettsiaceae members are connected by dotted lines. The ML whole-genome phylogeny was constructed using 55 single-copy orthologs (7,556 amino acids) extracted from the pangenome (JTT + I + G4m substitution model). The eight new *Rickettsia* genomes assembled in this study are indicated in bold and underlined. The *cif* pair phylogeny was inferred from 106 amino acids resulting from the concatenation of cifA and cifB (CPREV+G4 substitution model), and the tree is midpoint-rooted. For both phylogenies, bootstrap values were estimated from 1,000 replicates. Branch lengths represent number of amino acid substitutions per site. The number of *cif* pairs identified per genome is provided for each Rickettsiaceae member. *Cif* pairs are color-coded by type (V, VII, XI and X). The presence of additional uncertain *cif* pairs and their putative type is shown by the symbol ‘?’. *Rickettsia* isolate genomes associated with parthenogenesis (P), male-killing (MK) and cytoplasmic incompatibility (CI) are indicated.

As repeated lateral gene transfers have been identified as key drivers of *cif* distribution across *Wolbachia* strains [29–31,40,67], we extended the analysis to the Rickettsiaceae family, a group where *cif* pairs are abundant (Fig 5). Both evidence of horizontal transfers and cocladogenesis were apparent along the phylogenies of Rickettsiaceae and their *cif* pair (Mantel test, R = 0.69, *p* < 0.0001). The comparison of the Rickettsiaceae pangenome phylogeny with *cif* type distribution further revealed a patchy occurrence of *cif* pair types across six of the ten major *Rickettsia* groups (Spotted Fever, Scapularis, Transitional, Canadensis, Belli, and Rhyzobius), as well as in its sister genus *Tisiphia* and the basal Rickettsiaceae genera *Occidentia* and *Orientia* (Figs 5 and S7). While distinct strains within the same bacterial species often shared identical *cif* pair types (*e.g.*, *R. amblyommatis* strains and *O. tsutsugamushi* strains), other species exhibited polymorphism in *cif* pair presence or absence (*R. heilongjiangensis* strains, *R. felis* strains) or variation in *cif* pair types among strains (*R. hoogstraalii* CS strain with type V, RCCE3 strain with type X, and Croatia strain with no *cif* pair). In contrast, distantly related species could harbor highly similar, if not identical, *cif* pairs (*R. hoogstraalii* CS strain and *R. heilongjiangensis* TIGMIC strain), suggesting recent lateral *cif* transfers. These findings highlight a complex dynamic of *cif* pair losses and gains within the Rickettsiaceae family, mediated by both stable genomic associations through cladogenesis and horizontal gene transfers. This further suggests that different strains of the same bacterial species, differing for their *cif* pair, could commonly express distinct phenotypes.

**Fig 6:**
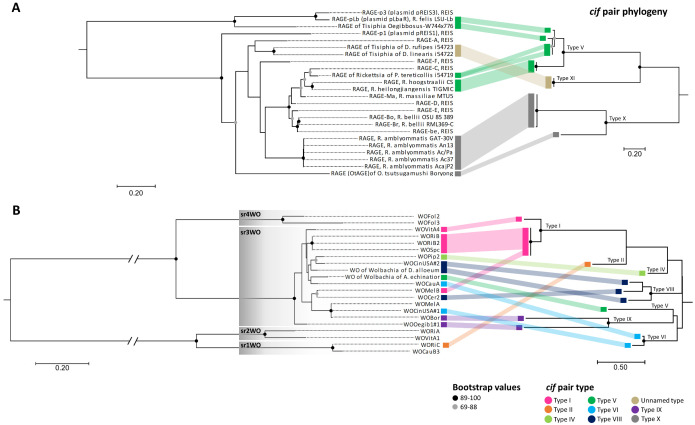
Phylogenetic comparison of the *cif* pair and their recurrent environments. The corresponding environment and *cif* pair phylogenies are connected and colored according to cif types. (A) RAGE and *cif* pair phylogenetic comparisons. The ML phylogeny of RAGE was constructed using five concatenated genes resulting in 681 amino acids (LG + G4 substitution model). The *cif* pair phylogeny associated was inferred from 164 amino acids resulting from the concatenation of *cifA* and *cifB* sequences (LG + G4m substitution model). (B) WO prophage and *cif* pair phylogenetic comparisons. The ML phylogeny of WO prophage is based on the large serine recombinase and constructed using 201 amino acids (FLU + G4 substitution model). The *cif* pair phylogeny associated was inferred from 164 amino acids resulting from the concatenation of *cifA* and *cifB* sequences (CPREV+G4m substitution model). The four WO clades (sr1WO, sr2WO, sr3WO and sr4WO) are indicated. All the trees are midpoint-rooted, and bootstraps were estimated from 1,000 replicates. Branch lengths represent number of amino acid substitutions per site.

Lateral transfers of *cif* pairs among unrelated bacteria were associated, in some cases but not all, with the concomitant transfer of the mobile genetic elements in which they are embedded. For instance, two distantly related *Rickettsia* species from distinct clades, *R. hoogstraalii* CS (Transitional group) and *R. heilongjiangensis* TIGMIC (Spotted Fever group), share identical *cif* pairs embedded within an identical 51,574 bp RAGE, suggesting that the entire RAGE was exchanged between these two bacteria (Figs 5 and S6). However, no correlation was found between the phylogenies of *cif* pairs and their associated RAGEs and WO prophages (Mantel test, R = 0.19 and 0.17, respectively, all *p* > 0.13), indicating that, although physically associated, RAGEs and WO prophages exhibit mostly independant evolutionary dynamics (Fig 6). This resulted in closely related *cif* pairs being associated with distantly related RAGEs (*e.g.*, for *cif* type V) or WO prophages (*e.g.*, for *cif* type I). However, in *Wolbachia*, the prophage srWO3 (shared between *Wolbachia* supergroups A and B) appears to serve as a preferential site for hosting *cif* pairs (Fig 6B) [41].

**Fig 7:**
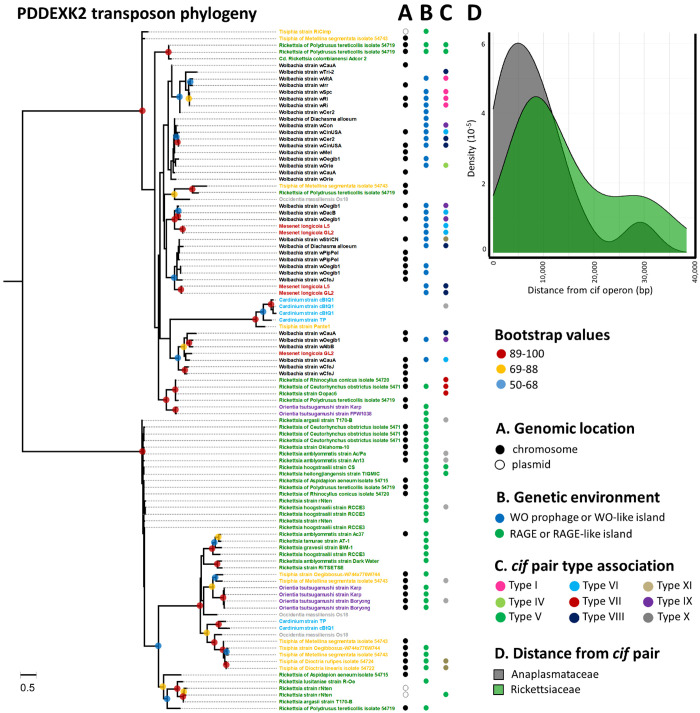
Phylogeny and genomic context of transposase-containing PD-(D/E)XK domain in bacterial genomes containing *cif* genes (ML, LG+ G4 substitution model, 189 amino acids). The tree is midpoint-rooted. Bootstrap values were estimated from 1,000 replicates. Branch lengths represent number of amino acid substitutions per site. Bacterial genera are color-coded as follows: black for *Wolbachia*, red for *Mesenet*, green for *Rickettsia*, yellow for *Tisiphia*, purple for *Orientia*, grey for *Occidentia* and blue for *Cardinium*. (A) Genomic location. Absence of circles indicates that the *cif* pair is located on an unlocalized contig. (B) Direct genetic environment. (C) *cif* type associated. (D) Density distribution of transposon distances from *cif* pairs in Anaplasmataceae and Rickettsiaceae. If multiple transposons were present near a *cif* pair, only the closest one was selected to ensure that each genome is represented by a single transposon.

Furthermore, the *cif* pairs were frequently associated with a specific group of transposons containing PD-(D/E)XK nuclease family transposase domains, known as PDDEXK2 transposases (Fig 7). These transposons were widespread across the *Wolbachia, Mesenet, Rickettsia, Occidentia, Orientia, Tisiphia*, and *Cardinium* genomes analyzed in this study, occurring in both main chromosomes and plasmids, within RAGE or WO regions. Phylogenetic analysis revealed two major clades, each comprising transposons from different symbionts, suggesting multiple horizontal transfer events both within and between bacterial families (Rickettsiaceae and Anaplasmataceae), as well as across genera (Fig 7). The majority of the *cif* pairs were found to be associated with these types of transposons, as evidenced by the presence of at least one PDDEXK2 transposon within a 40,000 bp region surrounding a *cif* pair in 54 out of 78 (69.2%) *cif*-containing genomic environments (Fig 7D). The median distance between these elements and *cif* pairs was 6,355 bp in Anaplasmataceae (*Wolbachia* and *Mesenet*) and 9,749 bp in Rickettsiaceae (*Rickettsia*, *Tisiphia*, and *Orientia*). A significant correlation was found between *cif* pairs and PDDEXK2 transposon phylogenies (Mantel test, R = 0.59, *p* = 0.0001), notably for *cif* types I and VII. However, this association was not absolute, as numerous PDDEXK2 transposons are devoid of *cif* pairs.

## Discussion

We analyzed 762 genomes of non-*Wolbachia* bacteria associated with arthropods, uncovering *cif* genes in 8.4% of cases, with a higher incidence of 17.35% in facultative symbionts. As expected, *Wolbachia* remains the predominant *cif* hotspot, harboring eight of the eleven identified types, but *cif* genes are far more widespread and diverse across bacterial lineages than previously assumed. Indeed, while *cif* genes were already known in non-*Wolbachia* bacterial strains [19,20,30,31,34,44], our extensive genome survey confirms and broadens this knowledge, revealing a more widespread pattern. *Rickettsia*, *Tisiphia, Mesenet,* and *Cardinium* also carry multiple *cif* types with apparently functional domain structures, while *Orientia*, *Occidentia*, *Rickettsiella*, and *Spiroplasma* each harbors one type. Beyond symbionts, *cif* genes were also present in several vertebrate pathogens, including human-infecting bacteria, but only in those also transmitted transovarially within arthropod hosts. In this context, although *Rickettsia* and *Orientia* are well-studied as vector-borne pathogens, their CI potential remains largely unexplored. Additionally, horizontal *cif* gene transfers occur not only within the *Wolbachia* genus [30,31] but also between distantly related bacteria, often within a genomic landscape shaped by mobile genetic elements. The presence of *cif* -containing bacteria in arthropod clades where *Wolbachia* is rare, such as ticks and other arthropod vectors, suggests that CI may be more common than previously thought.

### *Cif* genes in transovarially transmitted bacteria of arthropods

The large-scale analysis of arthropod-associated bacteria provides critical insights into the global distribution of *cif* genes and allows the identification of factors driving their spread. Indeed, their incidence in non-*Wolbachia* bacterial genomes is not random and results from a complex interplay between phylogenetic constraints and phenotypic determinants. The *cif* genes have been detected across a wide range of distantly related bacterial classes, but they are particularly prevalent in α-proteobacteria, especially within the Rickettsiales order. Rickettsiales are a group of obligate intracellular bacteria (with few exception [68,69]), primarily known for their ability to infect the cells of arthropods and mammals [69–72]. Interestingly, *cif* prevalence may be biased in certain *Rickettsiales* genera due to uneven whole-genome sequencing efforts across bacterial taxa, which have traditionally prioritized species of medical, veterinary, or agricultural importance. This is particularly evident in the case of *Rickettsia*, where sequencing has largely focused on pathogenic strains relevant to human health, thereby underrepresenting the broader diversity of non-pathogenic lineages [73,74]. However, as more non-pathogenic *Rickettsia* are sequenced, the number of *cif*-positive genomes within this genus is expected to rise. In this context, the current bias may actually result in an underestimation of *cif* prevalence in *Rickettsia*, and future sequencing of these underexplored lineages is likely to strengthen the trends observed here.

This unique intracellular lifestyle may thus make them a pivotal bacterial lineage where *cif* genes have proliferated and diversified into the 11 currently recognized types. The distribution of *cif* genes, however, is not fully explained by bacterial phylogeny alone. These genes are also concentrated in bacteria with specific phenotypic traits, particularly facultative symbionts and vertebrate pathogens that exhibit high rates of transovarial transmission in their arthropod hosts. These facultative symbionts may benefit disproportionately from the presence of *cif* genes and their ability to induce CI to enhance their transmission [1]. Yet, transovarial transmission alone does not fully account for the observed distribution. Notably, *cif* genes are absent from obligate symbionts, which play essential roles in host development and do not require CI to spread within arthropod populations. This expends what has been observed in *Wolbachia*, where obligate mutualists like *w*Cle in bed bugs and *w*Bm, *w*Ov, *w*Oo, and *w*Ppe in nematodes also lack the *cif* pair [30]. Broadly, this observation aligns with the hypothesis that endosymbionts tend to evolve toward mutualism over time [75], potentially through the loss of the *cif* pair.

The detection of *cif* genes in vector-borne pathogens such as *Orientia tsutsugamushi*, the causative agent of scrub typhus, and certain *Rickettsia* species like *R. heilongjiangensis*, responsible for Far Eastern spotted fever, is particularly intriguing. Traditionally, these pathogens are thought to persist primarily through horizontal (infectious) transmission between arthropod vectors and mammal hosts [70,71,74]. Vertical transmission within arthropod populations is believed to support their long-term maintenance, yet it is generally considered secondary to horizontal transmission [70,71,74]. The potential for these pathogens to exploit reproductive manipulation of arthropods as a drive system to enhance their spread among vector populations remains to be extensively investigated [44]. Considering this possibility could significantly reshape our understanding of the epidemiology of these vector-borne diseases. Indeed, these vector-borne bacteria should not be viewed solely as mammalian pathogens. Rather, they may occupy a dual role, as both vertebrate pathogens and vertically transmitted reproductive parasites in arthropods, as early pointed for *Rickettsia* [73]. In this context, vertical transmission coupled with reproductive parasitism may be key determinants of their prevalence in vector populations, while transmission to mammals could play a comparatively minor role in shaping their overall spread dynamics. Interestingly, early reports suggested that *O. tsutsugamushi* might manipulate the reproduction of Trombiculid mites, not through CI, but by inducing thelytokous parthenogenesis [76–78]. This observation raises the possibility that the *cif* genes could mediate both CI and parthenogenesis depending on the host, as supported by observations of parthenogenetic booklice infected with a *R. felis* strain carrying a plasmid containing *cif* genes [44]. However, reproductive manipulations are inherently associated with maternal inheritance, as it specifically benefits the reproduction of infected females. As a result, the occurence of *cif* genes in strictly horizontally transmitted pathogens is improbable. This likely accounts for the absence of *cif* genes in certain pathogenic *Rickettsia* species, such as *R. typhi* and *R. conorii*, as well as in *Anaplasma* and *Ehrlichia* species, which are close relatives of *Wolbachia* but seldom (if any) depend on transovarial transmission routes in their arthropod vectors [73,79].

### Polymorphism of *cif* gene domains

Our analysis reveals that the structure of the *cif* pair is widely conserved across the 11 *cif* types. For *Wolbachia* and eight other bacterial genera, this structure is well defined by the conservation of three key domains: the RNA-binding-like domain on *cifA*, the PD(D/E)-XK nucleases, and the AAA-ATPase-like domain on *cifB*, as primarily observed in *Wolbachia* [29,30,33]. This conservation has been well documented for *cif* type I-IV in *Wolbachia* [30], but remained understudied for types V-XI and non-*Wolbachia* bacteria [31,44]. These three domains are often accompanied by highly polymorphic additional domains, especially at the C-terminal position of *cifB*, reflecting ongoing dynamics of domain gain and loss. Although *cif* pairs of the same type generally exhibit similar domain structures, convergence can lead to similarities between distinct types, as observed among types V, VI, and VIII. Conversely, substantial polymorphism can arise within a single type, such as type X. Certain domains are consistently clustered together. For instance, RTX toxin domains always co-occur with DUF3491, while latrotoxin domains are frequently associated with ankyrin repeats. Ankyrin domains, alongside toxin modules like pore-forming TcdA/TcdB, RTX toxins, and latrotoxins, are likely to function as co-effectors that enhance the toxicity of PD-(D/E)XK nucleases by targeting additional components of arthropod cell [31]. Interestingly, the simplest *cifB* structure (comprising only the AAA-ATPase + PD(D/E)-XK nuclease, also called *cin* genes; [2]) is found in types II, III, IV, V, VIII, XI, and X. While types II-IV likely inherited this streamlined structure from a common ancestor [30], it remains unclear whether the other types acquired it from a convergence process through independent reduction or from the common ancestor of all *cifB*.

Most *cifA* and *cifB* genes are structurally intact and non-pseudogenized across the nine bacterial genera and all *cif* types, extending previous observations in *Wolbachia cif* types I-IV [30]. The well-conserved catalytic sites (D-E-K) in PD(D/E)-XK nucleases across 10 of the 11 *cif* types indicate that, despite domain polymorphism, most *cif* pairs could still induce CI through a conserved mechanism across bacterial genera, mirroring *Wolbachia*. It is worth noting that these exact catalytic sites, previously thought to be essential for DNase activity, are not always required to be intact to induce CI toxicity [36,61]. Morevover, recent findings confirm that CifA plays a more central role in CI induction in some systems as it carries a nuclear localization signal and exhibits DNase/RNase activity that induces CI-defining sperm defects prior to fertilization [36]. Other transgenic studies from different strains indicate that CifB alone can be the sole inducer of CI [38,39,61]. Additionally, certain *Rickettsia* strains harbor multiple *cif* pair copies, a feature commonly associated with strong CI in *Wolbachia* [29,46]. The hypothesis that the *cif* pairs function similarly across bacterial genera is supported by distributional, structural, and functional evidence, which suggest a functional conservation of *Wolbachia*’s CI mechanisms. However, empirical genotype-phenotype demonstrations between *cif* and CI are currently limited to *Wolbachia* types I, II and IV [28,29,36,38,39,47,80–82]. While CI phenotypes have been documented for bacteria harboring *cif* types III, V, VI, VIII, and IX [18–20,29], direct evidence linking the phenotypes to the activity of the *cif* pair is still lacking.

In *Rickettsiella* and *Cardinium*, the presence of single *cifB* homologs without loss-of-function mutations and no associated *cifA* raises questions about whether *cifB* serves functions beyond CI. In the context of CI, *cifB* is not expected to exist alone, as it should be pseudogenized and lost before *cifA* [30]; otherwise reproduction would be impaired due to self-incompatibility, unless *cifB* has acquired alternative functions such as acting in the autophagy pathway [83]. Moreover, the accessory *cifB* domains has been found in homologs that lack key CI effectors [42,44,84,85] (S2 Fig). Some of these elements induce male-killing, such as *spaid* in *Spiroplasma* and *oscar* in *Wolbachia* [86,87]. Additionally, *cifB* from *R. felis* LSU-Lb (type V) has also been proposed to induce parthenogenesis in booklice [44], though empirical evidence is still lacking. The distinction between *cif* genes and other toxins involved in male-killing or parthenogenesis may be more fluid and interconnected than previously thought [44,65]. The hypothesis of ‘multifaceted *cifB*’ could explain the dynamic gain and loss of their key domains, enabling bacteria to induce diverse phenotypes through a single gene depending on their arthropod host. This could explain cases like *Wolbachia w*CauA and *w*Bol1, where the phenotype shifts between CI and male-killing depending on the host species [88] or, within a single host species, on its genetic background [89].

### Dynamics of selfish *cif* pairs within the intracellular arena

While previous studies highlighted the mobility of the *cif* pair between *Wolbachia* strains [30,31,34,40,67], with occasional cases in *Rickettsiaceae* [20,34,44], our large-scale genomic analysis reveals a broader trend, occurring commonly within several bacterial genera but also, to a lesser extent, across distantly related genera. These findings support the hypothesis that the *cif* pairs function as selfish mobile elements [30], continuously jumping into new symbionts and evade potential extinction within ancient symbiont lineages, much like transposable elements that persist by spreading into new hosts [90]. The opportunity for coinfections, where different bacteria coexist within the same host [15,91], often in the same cell [92,93], along with the ability of facultative symbionts to switch between arthropod hosts [32,94,95], may have fostered the formation of freely recombining intracellular bacterial communities. This is postulated in the ‘intracellular arena’ hypothesis [96,97], which likely facilitated the transfer of *cif* pairs by promoting physical proximity between arthropod-associated bacteria, as observed for other mobile genetic elements [98].

How *cif* pairs jump between bacteria remains unclear, but they could potentially be exchanged via natural transformation or genetic vectors such as bacteriophages, plasmids, and transposons [99–102]. In *Wolbachia*, WO prophages, and particularly their EAM regions, are key drivers of *cif* pair diversification, facilitating genomic rearrangements and horizontal transfers between *Wolbachia* genomes [29,32,41,96,100]. We confirmed this pattern, and further evidenced that the *cif* pairs are more predominantly associated to sr3WO prophages than other WO phages. However, this WO-associated pattern is specific to *Wolbachia* and *Mesenet*, and does not apply to other bacteria harboring *cif* genes. In Rickettsiaceae, we find that distantly related *cif* pairs are often integrated within similar RAGE structures. Some authors previously found that the *cif* pairs in few Rickettsiaceae genomes were located near *Tra* genes of RAGE [34,44]. Our data further show that the *cif* RAGE-associated regions are often complete, with fully syntenic mobilization and cargo modules, or appear as RAGE-like islands (similar to WO-like islands in *Wolbachia*, [41]), as documented in other few genomes [62,64–66]. RAGEs are dynamic conjugative elements, that vary in copy numbers, undergoing genomic rearrangements, horizontally transferred, carrying mobile gene cargo [62,65,66]. This makes RAGEs similar to WO prophages, particularly in their sizes, which may explain why *cif* pairs have also diversified extensively within Rickettsiaceae. Many symbiosis-related genes, including those involved in male-killing, parthenogenesis, and symbiont densities, are carried by WO prophages [41,103,104], and RAGEs may carry similar genes [65]. Moreover, the transfer of RAGEs within plasmids have been observed in some cases [62,64], suggesting they could serve as vectors for *cif* gene transfers between different genomic compartments. In type V, RAGEs and plasmids likely synergized to drive *cif* proliferation among Rickettsiaceae, multiplying transfer mechanisms among insect, tick, and spider symbionts (S8 Fig).

SMGEs represent a third major mechanism driving genetic exchanges between *Wolbachia* strains and their associated WO prophages [31,40,105]. The presence of similar elements in *Rickettsiaceae* and their RAGEs suggests that SMGEs may also play a crucial role in the spread and proliferation of *cif* pairs across a much broader phylogenetic range. Indeed, we identified the PDDEXK2 transposase, previously recognized as a frequent neighbor of *cif* pairs in *Wolbachia* genomes [31], also in *Mesenet*, *Rickettsia*, *Occidentia*, *Orientia*, *Tisiphia*, and *Cardinium*. The PDDEXK2 transposase undergoes lateral transfer across different bacterial genera and exhibits a co-phylogenetic signal with the *cif* pair, suggesting it could be a key driver of *cif* lateral transfers through co-transmission. Alternatively, SMGEs can integrate into WO prophages and RAGEs or proliferate within bacterial chromosomes or plasmids, potentially accumulating in transposon- and retrotransposon-rich islands. These islands may harbor *cif* pairs, a pattern previously observed in *Wolbachia* [105], but also commonly found in other symbionts. However, exceptions exist, as seen in *Rickettsiella*, where no association of their *cif* pairs with SMGEs was observed, a pattern consistent with the relatively few mobile elements identified in their genomes [106].

As a result, the mechanisms governing *cif* genes lateral transfers are likely multifaceted and synergistic. The prevailing pattern reveals a consistent association between *cif* pairs and mobile genetic elements of varying scales, from small SMGEs to large intact prophages and RAGEs. Although exceptions exist, the typical *cif* pair duplication mechanism seems to follow these steps: (i) the *cif* pair is flanked by SMGEs (often a PDDEXK2 transposase) within a repetitive environment such as WO or RAGE; (ii) the SMGEs and *cif* pair jump into the genome of the same or a new bacterium within the host intracellular arena, reinserting into a structurally similar repetitive environment; (iii) large rearrangements or transfert of WO or RAGE can also copy the *cif* pair and facilitate dynamic domain exchanges. This continuous reshuffling of *cif* pairs may generate substantial genetic diversity, with the fittest variants thriving if they offer a selective advantage to the bacteria. This dynamic parallels the spread of antibiotic resistance genes via mobile genetic elements, highlighting convergent mechanisms of bacterial adaptation [107,108].

## Conclusion

The presence of *cif* pairs in arthropod-associated intracellular bacteria beyond *Wolbachia* carries significant evolutionary, ecological, and applied implications. Notably, potential CI-inducing bacteria are widespread in arthropod groups where *Wolbachia* is rare, including key vector groups like ticks. While *Wolbachia* is uncommon in ticks [109–111], maternally inherited *Rickettsia* are frequent and can reach near-fixation, aligning with expectations for a CI-driven dynamic [109,110,112–114]. This suggests certain *Rickettsia* strains may shape tick biology much like *Wolbachia* influences mosquitoes, highlighting their potential for vector control strategies. Beyond ticks, *Rickettsia* is also more prevalent than *Wolbachia* in other chelicerates, *Trombidiformes*, and *Coleoptera* [17,32,115], suggesting an even broader impact in major arthropod groups. Additionally, the discovery of mobile genetic elements enabling gene exchange between *Wolbachia* and other intracellular bacteria opens new avenues for genetic manipulation. The inability to modify *Wolbachia* has been a major limitation, but harnessing these elements could revolutionize biotechnology and vector control by allowing precise genetic edits. Engineering *Wolbachia* and other symbionts could also provide deeper insights into host-symbiont interactions, reproductive manipulations like cytoplasmic incompatibility, and the broader evolutionary dynamics of symbiosis.

## Materials and methods

### Genome assembly of new *Rickettsia* strains isolated from ticks

Tick specimens of *Ornithodoros erraticus* (soft ticks, Argasidae), *Ixodes arboricola*, *Dermacentor reticulatus*, and *Amblyomma dissimile* (hard ticks, Ixodidae) were collected in Europe or French Guiana (S4 Table). For all tick specimens, we obtained genomic DNA (gDNA) extractions enriched in endosymbiont DNA after the dissection and a 5-µm filtration of the Malpighian tubules and ovaries (for adult females), two organs highly enriched in endosymbionts [116,117]. This was performed following a previously described protocol [117,118] (see S4 Table for pooling details and DNA extraction methods). For each sample, the resulting enriched gDNA was quantified on Qubit using the dsDNA high-sensitivity kit (Invitrogen).

Metagenomes of filtered organs were sequenced using different technologies depending on the sample (see S4 Table for details). Seven out of eight metagenomes were sequenced with short-read Illumina technologies, either on the HiSeq 2500 platform with the Nextera DNA Sample Preparation Kit, or on the NovaSeq 6000 platform with the NovaSeq Reagent Kits. For the *O. erraticus* metagenome, reads were assembled using MEGAHIT (v1.2.9) [119]. To retrieve *R. lusitaniae* strain R-Oe genome, meta-assembled contigs were binned using CONCOCT (v1.1.0) [120] and taxonomic assignment was performed with the Anvi’o pipeline (v7.1) [121]. To reduce the redundacy and obtain the final *R. lusitaniae* strain R-Oe genome, we BLAST all contigs against the NCBI database [122], those that did not show sequence similarity with any *Rickettsia* representative were removed. For the metagenomes of *I. arboricola*, *D. reticulatus*, and three out of four *A. dissimile* samples, *Rickettsia* genome processing followed a modified version of the Blobology pipeline [123] available on GitHub [124]. Contaminating sequences were filtered out, and assemblies manually refined by inspecting assembly graphs with Bandage (v0.8.1) [125]. The quality and completeness of the seven first *Rickettsia* genome assemblies were assessed using QUAST (v4.6.3) [126] and miComplete (v1.1.1, -hmms Bact105) [127]. Sequencing of one metagenome of *A. dissimile* was performed on a MinION device using a R9.4.1 flow cell (Oxford Nanopore technology), with library preparation via the Ligation Sequencing Kit SQK-LSK109. Basecalling was performed with Guppy (v4.2.2) (Oxford Nanopore Technologies) in high accuracy mode and basecalled reads were trimmed using Porechop (v0.2.4) [128]. Metagenome assembly was performed using Flye (v2.9) [129,130], and consensus contigs generated using Medaka (v1.2.2) [131]. Genome completeness was assessed using miComplete (v1.1.1, -hmms Bact105) [127] and the BUSCO (v5.3.2) pipeline [132].

Accordingly, we de novo assembled eight genomes from four tick species: (i) *Rickettsia lusitaniae* strain R-Oe isolated from *Ornithodoros erraticus*, (ii) *R. vini* strain IarboMS4 isolated from *Ixodes arboricola*, (iii) *R. raoultii* strains Dreti_100P and Dreti_100F isolated from *Dermacentor reticulatus*, and (iv) *Rickettsia sp.* strains AdisF19, AdisM1, AdisP2, and AdisP3Sn isolated from *Amblyomma dissimile* (Table 1 and Figs 5B and S9).

### Public genome collection and annotation

Assembled genomes from public databases were collected for a representative set of ‘non-*Wolbachia*’ maternally inherited symbionts and their close relatives. This set includes 31 bacterial genera listed in S1 Table. The genome search was conducted as exhaustively as possible using the GenBank database [122], including symbiont genomes assembled from host eukaryote genome sequencing data from the Darwin Tree of Life project [133] or published articles [134,135]. As references, we also included 24 representative genomes of *Wolbachia* known to harbor specific *cif* genes from types I to IX [30,31,34]. In total, 778 public genomes were retrieved, including 754 non-*Wolbachia* and 24 *Wolbachia* genomes. All the genomes used in this study were then annotated using Prokka (v1.14.6) [136] with adapted genetic code depending on bacterial genera. For each genus, the Average Nucleotide Identity (ANI) was determined by performing an all-vs-all genome comparison using fastANI (v1.33) [137]. The completness pourcentage of each genomes were estimated using BUSCO (v.5.5.0) with the database ‘bacteria_odb10’ [132].

### *Cif* genes screening and prediction of functional domain compositions

To determine the presence of *cif* genes in the genomes, we used Orthofinder (v2.5.4) [138] to search for specific orthologs of both *cifA* and *cifB* genes independently. We employed a curated amino acid database of *cifA* and *cifB* queries, initially created using *Wolbachia* and *Rickettsia cifA* and *cifB* sequences from Martinez et al. [30] as reference. To assess the efficiency of our reference queries, we first analyzed genomes known to harbor *cifA-cifB* pairs [30,34,44]. We then extended the analysis to the remaining genomes. Each identified orthogroup matching *cifA* or *cifB* was examined to distinguish true from false positives. To this end, we first aligned each matched orthogroup with *cifA* or *cifB* reference sequences using MAFFT (v7.505) [139] via the EMBL European Bioinformatics Institute website [140]. Only sequences that aligned with reference *cifA* or *cifB* were retained for further exploration of their protein domains (see below). *cifB* sequences were considered true positive only if they harbored a PD-(D/E)XK nuclease domain (see below) and were coupled with an upstream *cifA*. However, in cases where no upstream *cifA* was detected but the PD-(D/E)XK nuclease domain was present, the corresponding ORFs were classified as hypothetical *cifB*. In these instances, additional domain analyses and phylogenetic consistency were required to confirm their identity (examples of ORFs that aligned with reference *cifB* but did not meet the established criteria are shown, S2 Fig). Once novel *cifA* or *cifB* sequences were confidently identified, they were added to the queries’ database, and new OrthoFinder analyses were performed on the annotated genomes, followed by detail examination for confirmation. The final database of *cifA* and *cifB* queries is available on GitHub (see Data availability section).

Protein domains were predicted for *cifA* and *cifB* homologs using the HHpred webserver [141] using default parameters. Amino acid sequences were individually queried against the following databases: SCOPe70 (v.2.08), Pfam-A (v37.0), SMART (v6.0), and COG/KOG (v1.0). Only hits with a probability of >50% were retained. In some cases, specific predicted domains in one sequence were not detected by HHpred in closely related *cif* homologs, despite their expected presence. To address this, we also performed independent alignments of all domain sequences across all *cifA* or *cifB* gene sequences using Clustal Omega [142]; the domain database is available on GitHub. Visualization of protein domains within *cif* genes was conducted using RStudio (v4.3.1) with an in-house script, employing the ggplot2 (v3.4.4) [143], cowplot (v1.1.1) [144], and gridExtra (v2.3) [145] packages.

### Manual research and reconstruction of *cif* disrupted by mutations

Some Prokka annotations of *cifA* or *cifB* resulted in incomplete or multiple successive ORFs rather than a single and continuous ORF per gene. This pattern suggested that an ancestral full-length ORF may have been fragmented by loss-of-function mutations, potentially leading to pseudogenization. To explore this possibility, we retrieved the nucleotide sequences encompassing all ORFs annotated as *cifA* or *cifB*, and manually translated them into amino acids in all three reading frames using the ExPASy translation tool [146]. The positions of premature stop codons and frameshifts were recorded allowing to localize and count the disruptive mutation event(s) on each genes. As an exploratory approach, we then replaced disruptive mutations with non-disruptive codons by substituting stop codons with sense codons in order to reconstruct, when feasible, a hypothetical uninterrupted ORF. To verify the accuracy of the reconstructed ORFs, the amino acid sequences of disrupted *cifA* or *cifB* were aligned with closely related complete *cifA* or *cifB* genes. This allowed us to reveal previously hidden protein domains that would have been missed due to gene fragmentation. The reconstructed sequences were used in downstream analyses, as they provided a more accurate representation of the original coding regions prior to pseudogenization. This enabled us to characterize conserved domains, evaluate the functional potential of fragmented genes, and reduce biases introduced by automated gene prediction errors.

### Characterisation of the *cif* pair environments

By default, Prokka enables an initial annotation step by consistently identifying common bacterial-associated genes and small mobile elements, such as IS transposons. However, Prokka relies on a limited database for endosymbiont bacteria, and most predicted ORFs are labeled as ‘hypothetical proteins,’ making it suboptimal for detecting prophages or specific Rickettsiaceae-associated genes. To overcome this limitation, annotated genomes carrying *cif* genes were screened again custom databases of RAGE and WO prophage genes using Orthofinder. The RAGE database was constructed using F- and Ti-like T4SS genes and their flanking regions from *Rickettsia* sp. strain REIS, *R. bellii* strains RML369-C and OSU 85–389, *R. massiliae* strain MTU5, *R. peacockii* strain Rustic, and *R. felis* strain LSU-Lb as references [62,64] (S5 and S6 Tables). The WO prophage database was compiled from both core-prophage genes and their eukaryotic association modules (EAMs) from 19 representative WO prophages or WO-like islands, as described by [41] (S5 and S7 Tables). RAGE or WO genes located near *cif* pairs were identified accordingly. These Orthofinder searches enabled the characterization of most ORFs surrounding *cif* pairs. However, some genes did not match any database entries and remained labeled as ‘hypothetical proteins.’ In such cases, BLASTP searches [147] were performed using default parameters, with successful matches (>70% query coverage and percentage identity). If BLASTP identified an ORF encoding for Ankyrin repeats, further validation was conducted using HHpred and SMART [148], as Ankyrin repeat annotations from BLASTP are prone to false positives. Finally, an in-house R script was used to visualize gene environments, utilizing the ggplot2, dplyr (v1.1.4) [149], and stringr (v1.5.1) [150] packages.

The presence of the specific transposon containing a PD-(D/E)XK nuclease family transposase domain, first described by Tan et al. [31], was investigated in all *cif*-positive genomes using OrthoFinder with queries provided in that same study. Conservation of the PD-(D/E)XK nuclease family transposase domain was checked with HHpred using the same databases as for *cifA* and *cifB*. Some of these transposons were located near *cif* pairs. In such cases, the distance to the nearest *cifA* or *cifB* was measured in base pairs.

### Phylogenetic and statistical analysis

All phylogenetic analyses conducted in this study were performed on amino acid sequences and the maximum likelihood (ML) method with 1,000 bootstrap replicates. The most suitable evolutionary model was determined using modeltest-ng (v0.1.7) [151] based on the corrected Akaike Information Criterion (AICc), and ML trees were inferred using raxml-ng (v1.1.0) [152]. The phylogenies of the different *cif* pairs were constructed using three conserved domains (RNA-binding-like, AAA-ATPase-like, and PD-(D/E)XK nucleases), which were independently aligned using MAFFT and manually concatenated. Single *cifB* phylogenies were also generated using concatenated sequences of the AAA-ATPase-like and/or PD-(D/E)XK nucleases. The Rickettsiaceae family phylogenetic relationship was determined using a whole-genome phylogeny. Single-copy orthologs (SCOs) from Rickettsiaceae members were identified using OrthoFinder. These SCOs were individually aligned using MAFFT, and ambiguous regions were subsequently removed using trimAl (v1.4.1) [153] prior to concatenating the individual alignments with an in-house Python script. The RAGE phylogeny was assessed using a concatenation of five shared genes (*TraU, TrbC, TraN, TraF,* and *TraD*). The WO prophage phylogeny was constructed using the large serine recombinase WO (*WD_0288* for WOMelA and *WD_0634* for WOMelB in the wMel reference genome: AE017196.1), as described by Bordenstein and Bordenstein [41]. The *Orientia* network analyses were made using Splitstree [154].

All statistical analyses were carried out using RStudio [155]. Multiple comparisons between the different variables were performed using both parametric and non-parametric tests. PERMANOVA test was made with the vegan (v.2.6.8) [156] package. For Chi-square test, p-values were estimated using a Monte Carlo simulation-based Chi-square test (B = 10,000) due to low expected counts in some categories. For the environments of *cif* pairs, two levels of analysis were performed: (i) the overall categorical structure (WO, RAGE, SMGE or other) and (ii) the phylogenetic association. The congruence between the compared phylogenies was tested using Mantel tests on the pairwise patristic distances between sequences, employing Spearman methods and 9,999 permutations, with the vegan, ape (v.5.8) [157], and phytools (v.2.4.4) [158] packages. To minimize the potential for sampling bias due to redundancy, only one sequence per species was included in the analysis.

## Supporting information

S1 Table Accession number and main informations of the genomes used in this study. The eight newly assembled *Rickettsia* genomes are indicated in bold and underlined.
(XLSX)

S2 Table Details of *cif* pair characteristics. The absence (0), presence (1) or unknown information because of end of contigs (NA) for each protein domain is indicated.
(XLSX)

S3 Table Summary of the total number of *cifA-cifB* genes detected and domain presence by genus. (*) Single *cifB* gene present without an associated *cifA* gene. The *Wolbachia* genomes screened here are limited in number and do not represent the full diversity of available *Wolbachia* genomes.
(XLSX)

S4 Table Informations about tick specimens, pooling, DNA extraction and sequencing methods that allowed the assembly of the eight new *Rickettsia* genomes. (XLSX)

S5 Table Accession number and main informations of the referenced RAGEs and WO prophages used to create the RAGE and WO prophage genes databases. (XLSX)

S6 Table Details of the RAGE database and the corresponding CDS on the *Rickettsia* genomes annotated with Prokka. (XLSX)

S7 Table Details of the WO prophage database and the corresponding CDS provided in GenBank. (XLSX)

S1 Fig Phylogenies and domain composition of all the *cifB* genes analyzed in this study. The additional *cifB* genes not represented in Fig 2, from *Cardinium*, *Orientia*, *Rickettsia*, and *Rickettsiella*, are highlighted in bold. (A) ML phylogeny of the AAA-ATPase-like and PD-(D/E)XK nuclease N-terminal domains, concatenated (JTT-DCMUT+G4 substitution model, 86 amino acids). (B) ML phylogeny of the PD-(D/E)XK nuclease N- and C-terminal domains, concatenated (JTT + G4 substitution model, 92 amino acids). ‘○’ indicates *cifB* genes without any associated *cifA*. The trees are midpoint-rooted, and bootstrap values were estimated from 1,000 replicates. The cif pair types (I–X) are grouped according to the classification in Fig 2. Open reading frame (ORF)-disrupting mutations are indicated by black vertical lines.
(TIF)

S2 Fig Relation between protein domain composition, lenght and *cif*-like gene nature. (A) Correlation between the total *cif* pair length and the number of predicted domains (Adjusted R^2^ = 0.98, *p* = 5.10^-16^; predicted by a polynomial regression on LOESS values). (B) Polymorphism of protein domains in the *cif* pair and other genes with cif domains. The associated phenotypes are indicated: Cytoplasmic Incompatibility (CI) and Male-killing (MK). According to our knowledge, the genotype-phenotype link has only been empirically demonstrated for the CI deubiquitinase genes, called *cid* (type I), and the CI nuclease genes, called *cin* (type II and IV), but *cif* types V, VIII, IX, X and XI share a similar protein domain composition with *cin* genes. Asterisks indicate that the CI phenotype is known, but the genes and domains implicated have not been tested, while *cif* genes are strongly suspected. Some polymorphic ORFs were identified containing protein domains shared with other *cifB* genes but lacking an upstream *cifA* and/or a PD-(D/E)XK nuclease phylogenetically related to other *cifB* genes. These ORFs were considered as other genes distinct from *cifA-cifB*, but sharing some similar domains.
(TIF)

S3 Fig Independent ML phylogenies of each protein domain identified in *cifA* and *cifB.* Ankyrin repeat domains were too polymorphic to be consistently aligned together. (A) Apoptosis regulator-like (35 amino acids, JTT + G4 substitution model). (B) RNA-binding-like (62 amino acids, CPREV+G4 substitution model). (C) AAA-ATPase-like (47 amino acids, CPREV+G4 substitution model). (D) PD-(D/E)XK nuclease (140 amino acids, CPREV+G4cd substitution model). (E) OTU-like cysteine protease (165 amino acids, FLU + G4 substitution model). (F) Deubiquitinase DUB (68 amino acids, FLU + G4 substitution model). (G) Pore forming toxin TcdA/B (114 amino acids, JTT + G4 substitution model). (H) DUF3491 (104 amino acids, FLU + G4 substitution model). (I) RTX toxin (50 amino acids, FLU + G4 substitution model). (J) Salivary-gland toxin (104 amino acids, JTT + I substitution model). (K) Latrotoxin (64 amino acids, HIVB+G4 substitution model).
(TIF)

S4 Fig Amino acid alignment of the PD-(D/E)XK nuclease from C-terminal (A) and N-terminal (B). The three key catalytic residues ‘D–E–K’ are highlighted in red. The *cif* types I-X are indicated.
(TIF)

S5 Fig Gene syntenic environments of the cif pairs in additionnal non-*Wolbachia* genomes. (A) Environment composed of small mobile genetic elements (SMGE) or RAGE-like. The yellow asterisk highlights homologous *cif* syntenic environments inherited through cladogenesis. (B) Environment devoid of mobile elements. (C) Environment interpretation limited by excessively small contigs. Gene lengths are not to scale. *Cif* type V, VII, XI and X are indicated below the bacterial strain names.
(TIF)

S6 Fig Transfer, duplication and rearrangements of large block region inside and between *Rickettsia* genomes. (A) Adjacent duplication of an intra-RAGE region flanked by two integrases. Gray dashed lines represent the continuation of the genome onto the next line for visualization purposes. (B) Plasmids densely covered with small mobile elements showing genomic rearrangements. (C) Identical RAGE structure identified on contigs of two unrelated *Rickettsia* strains. Their corresponding phylogenetic groups are indicated in grey. Position coordinates of the six represented genomic regions of the *Rickettsia* endosymbionts are indicated in parentheses. Gray lines connect orthologous genes. The gene delimitation lengths are relative to the indicated scale.
(TIF)

S7 Fig Phylogenetic and network analysis of 23 *Orientia tsutsugamushi* strains: AFSC4 (OriASFSC4), AFSC7 (OtsuAFSC7), Boryong (OtsuBor), FPW1038 (OtsuFPW1038), Gilliam (OriGil), Ikeda (OtsuIke), JJOtsu1 (OriJJ1), JJOtsu5 (OriJJ5), JJOtsu6 (OriJJ6), JJOtsu7 (OriJJ7), JJOtsu8 (OriJJ8), Karp (OtsuKarp), Kato (OriKato), Sido (OriSido), TA686 (OriTA686), TA716 (OriTA716), TA763 (OriTA763), TW-1 (OriTW1), TW-22 (OriTW22), UT144 (OtsuUT144), UT176 (OriUT176), UT76 (OriUT76), and Wuj/2014 (OriWuj). (A) Whole-genome phylogeny of the two *Orientia* species: *O. tsutsugamushi* and *O. chuto* (OriDubai). *Occidentia massiliensis* Os18 (OmasOs18) and *Cd.* Megaira MegNEIS296 (CdMegNEIS) were used as outgroups. The maximum-likelihood (ML) whole-genome phylogeny was constructed using 95 single-copy orthologs (SCO) (26,928 amino acids) extracted from the pangenome (CPREV+G4m substitution model). (B) Network analysis of the 95 concatenated SCOs using the Neighbor-Net method. Each edge (or set of parallel edges) represents a split in the dataset, with its length corresponding to the split’s weight. Clades supported by bootstrap values >89 are colored. (C) Network analysis of the *cif* pair from *O. tsutsugamushi* concatenated from 1,551 amino acids. The corresponding clade colors of strains carrying the *cif* pairs are represented. (D) Alignment of the 1,551 amino acids of the *cifA-cifB* pair. Protein domain locations are highlighted, corresponding to the colors used in Fig 2 (purple: RNA-binding-like; green: AAA-ATPase-like; red: PD-(D/E)XK nuclease). Residue conservation along the cif pair is shown by the height of each peak: solid peaks indicate high conservation, while fragmented peaks indicate low conservation at these positions.
(TIF)

S8 Fig Proposed ‘gain-and-loss’ scenario for the diversification of *cif* type V in the Rickettsiaceae family, in close association with RAGE environments among arthropods. This scenario outlines how type V *cif* genes may have spread within Rickettsiaceae through an ancestral *cif* pair in a clade 1 RAGE module. (1) Horizontal transfer of a RAGE drove *cif* pair exchange between two unrelated *Rickettsia* species, possibly during co-infection in a same host. (2) In *Rickettsia* infecting the beetle *Polydrusus tereticollis*, a chromosomal region containing *cif* was duplicated within a RAGE, inherited from the ancestral clade 1 RAGE. (3) This *cif* pair, or a close relative, transferred into a plasmid of *r*Nten, a *Rickettsia* infecting *Nesidiocoris tenuis*, via small mobile genetic elements (SMGEs), then moved to a second plasmid. (4) A type V *cif* pair was likely present in the chromosomal RAGE-C of the *Rickettsia* endosymbiont of *Ixodes scapularis* (REIS) before pseudogenization, possibly due to acquiring the biotin operon (vitamin B synthesis [62]), shifting its role from reproductive manipulator to nutritional mutualist. Previously, the *cif* pair may have proliferated between genomic compartments and moved onto the pREIS-3 plasmid within a clade 2 RAGE (RAGE-p3). (5) This clade 2 RAGE and its *cif* pair, or a close relative, transferred into a plasmid of another *Rickettsia* species infecting booklice (*Liposcelis bostrychophila*, *Rickettsia felis* strain LSU-Lb), then integrated into the chromosome of a *Tisiphia* infecting the spider *Oedothorax gibbosus* (Oegibbosus-W744x776). (6) In REIS, the RAGE-p3 has lost its putative *cif* pair. Open-source images available are used under permissive licenses from Openclipart (https://openclipart.org/share) and Pexels (https://www.pexels.com/license/).
(TIF)

S9 Fig Genome maps of the eight *Rickettsia* genomes assembled in this study: *Rickettsia vini* strain IarboMS4 (A), *R. raoultii* strain Dreti_100P (B) and Dreti_100F (C), *R. lusitaniae* strain R-Oe (D), *Rickettsia* sp. strain AdisF19 (E), AdisM1 (F), AdisP2 (G) and AdisP3Sn (H). Circles on genome maps correspond to the following (from the edge to the middle): (1) forward strand genes; (2) contigs in dark and light gray; (3) GC contents; (4) reverse strand genes. In grey, genomes from the Spotted Fever Group (SFG); in pink, from the Transitional Group (TRG); in green, from the Belli Group (BEL).
(TIF)

S10 Fig Whole-genome phylogeny of the *Rickettsiella* genus based on 102 single-copy orthologs (SCO) (ML, LG+ I + G4 substitution model, 18,951 amino acids). Genomes of *Aquicella, Coxiella, Legionella*, and *Berkiella* were used as outgroups. Bootstrap values were estimated from 1,000 replicates. (A) Presence of *cif* genes, with colors referring to *cif* type IX. (B) Host order from which the genome was isolated.
(TIF)
