# Supplementary figures and images for: Diversity and spread of cytoplasmic incompatibility genes among maternally inherited symbionts
Source: PLoS Genet. 2025 Sep 9;21(9):e1011856. doi: 10.1371/journal.pgen.1011856 (PMC12445526; doi:10.1371/journal.pgen.1011856)

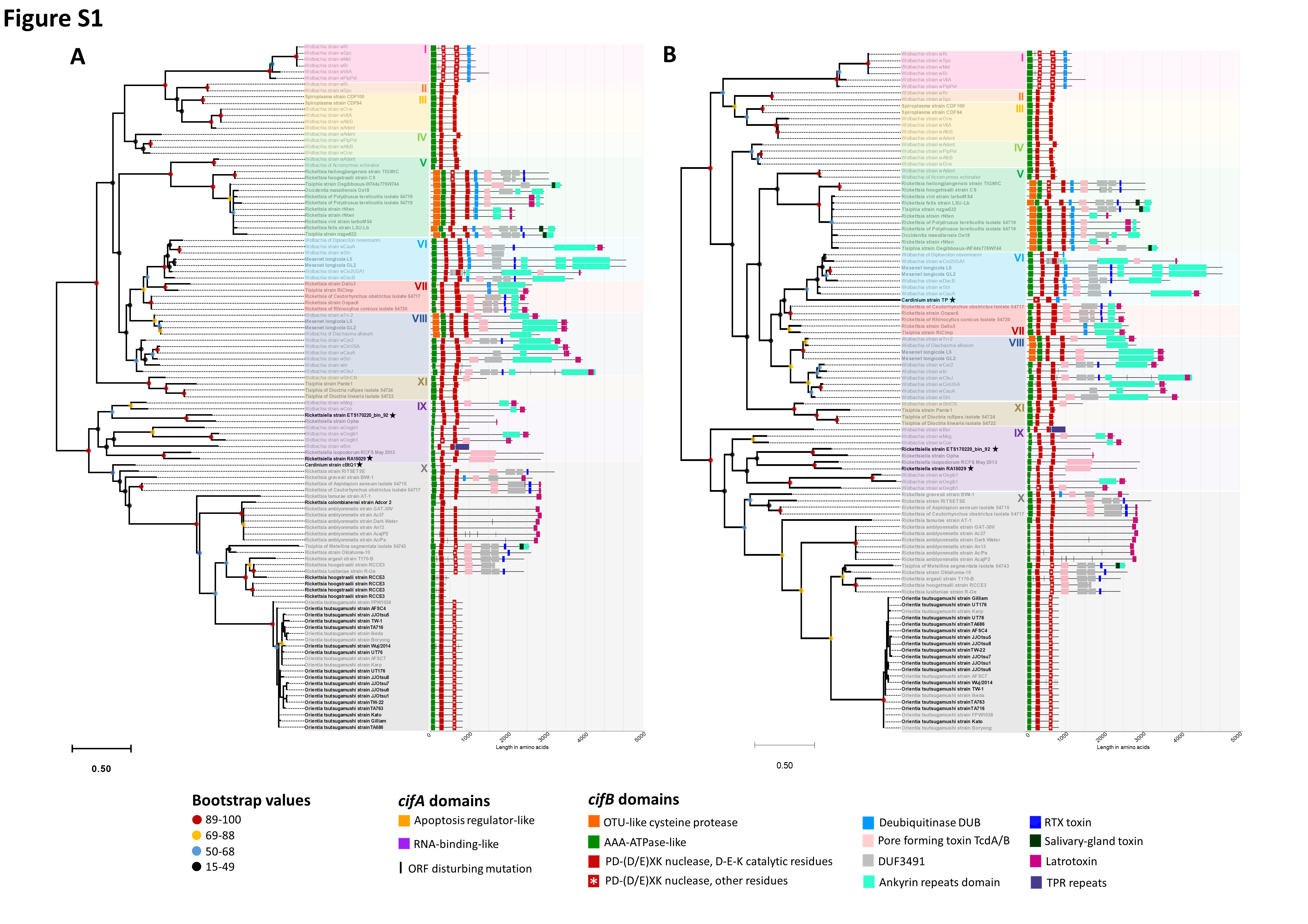

Supplement: S1 Fig — The additional cifB genes not represented in Fig 2, from Cardinium, Orientia, Rickettsia, and Rickettsiella, are highlighted in bold. (A) ML phylogeny of the AAA-ATPase-like and PD-(D/E)XK nuclease N-terminal domains, concatenated (JTT-DCMUT+G4 substitution model, 86 amino acids). (B) ML phylogeny of the PD-(D/E)XK nuclease N- and C-terminal domains, concatenated (JTT + G4 substitution model, 92 amino acids). ‘○’ indicates cifB genes without any associated cifA. The trees are midpoint-rooted, and bootstrap values were estimated from 1,000 replicates. The cif pair types (I–X) are grouped according to the classification in Fig 2. Open reading frame (ORF)-disrupting mutations are indicated by black vertical lines. (TIF) [file pgen.1011856.s008.TIF]

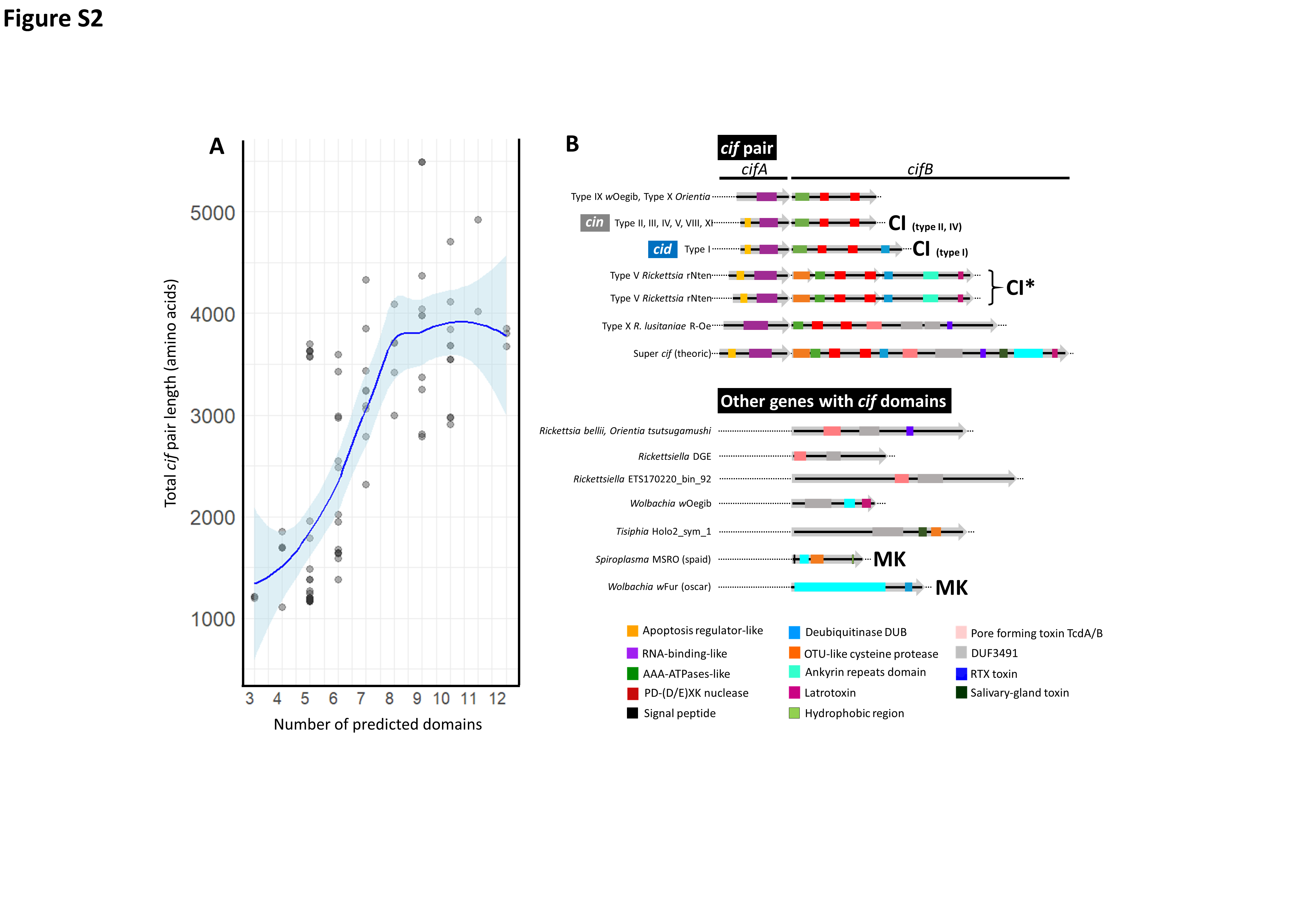

Supplement: S2 Fig — (A) Correlation between the total cif pair length and the number of predicted domains (Adjusted R2 = 0.98, p = 5.10-16; predicted by a polynomial regression on LOESS values). (B) Polymorphism of protein domains in the cif pair and other genes with cif domains. The associated phenotypes are indicated: Cytoplasmic Incompatibility (CI) and Male-killing (MK). According to our knowledge, the genotype-phenotype link has only been empirically demonstrated for the CI deubiquitinase genes, called cid (type I), and the CI nuclease genes, called cin (type II and IV), but cif types V, VIII, IX, X and XI share a similar protein domain composition with cin genes. Asterisks indicate that the CI phenotype is known, but the genes and domains implicated have not been tested, while cif genes are strongly suspected. Some polymorphic ORFs were identified containing protein domains shared with other cifB genes but lacking an upstream cifA and/or a PD-(D/E)XK nuclease phylogenetically related to other cifB genes. These ORFs were considered as other genes distinct from cifA-cifB, but sharing some similar domains. (TIF) [file pgen.1011856.s009.TIF]

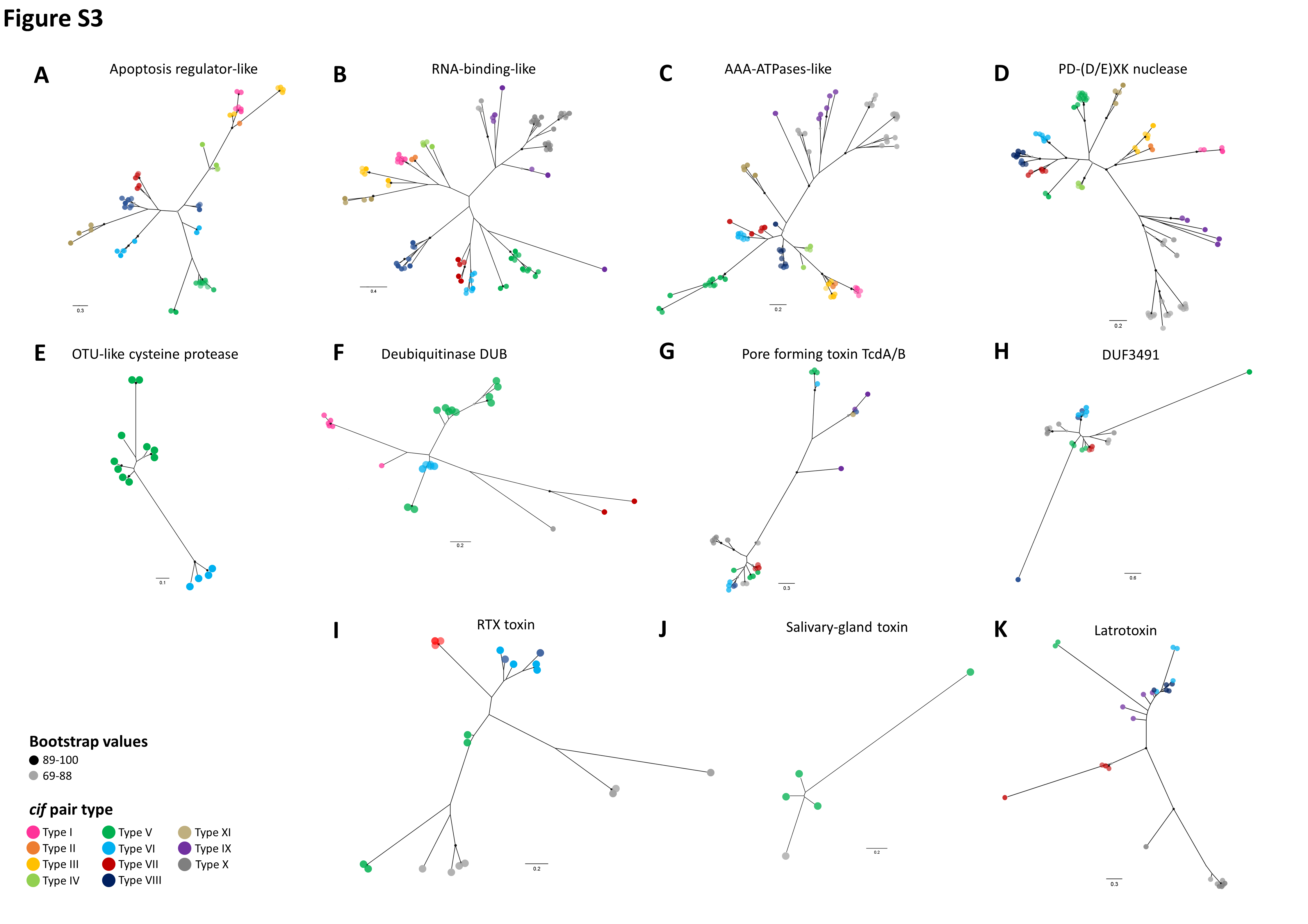

Supplement: S3 Fig — Ankyrin repeat domains were too polymorphic to be consistently aligned together. (A) Apoptosis regulator-like (35 amino acids, JTT + G4 substitution model). (B) RNA-binding-like (62 amino acids, CPREV+G4 substitution model). (C) AAA-ATPase-like (47 amino acids, CPREV+G4 substitution model). (D) PD-(D/E)XK nuclease (140 amino acids, CPREV+G4cd substitution model). (E) OTU-like cysteine protease (165 amino acids, FLU + G4 substitution model). (F) Deubiquitinase DUB (68 amino acids, FLU + G4 substitution model). (G) Pore forming toxin TcdA/B (114 amino acids, JTT + G4 substitution model). (H) DUF3491 (104 amino acids, FLU + G4 substitution model). (I) RTX toxin (50 amino acids, FLU + G4 substitution model). (J) Salivary-gland toxin (104 amino acids, JTT + I substitution model). (K) Latrotoxin (64 amino acids, HIVB+G4 substitution model). (TIF) [file pgen.1011856.s010.TIF]

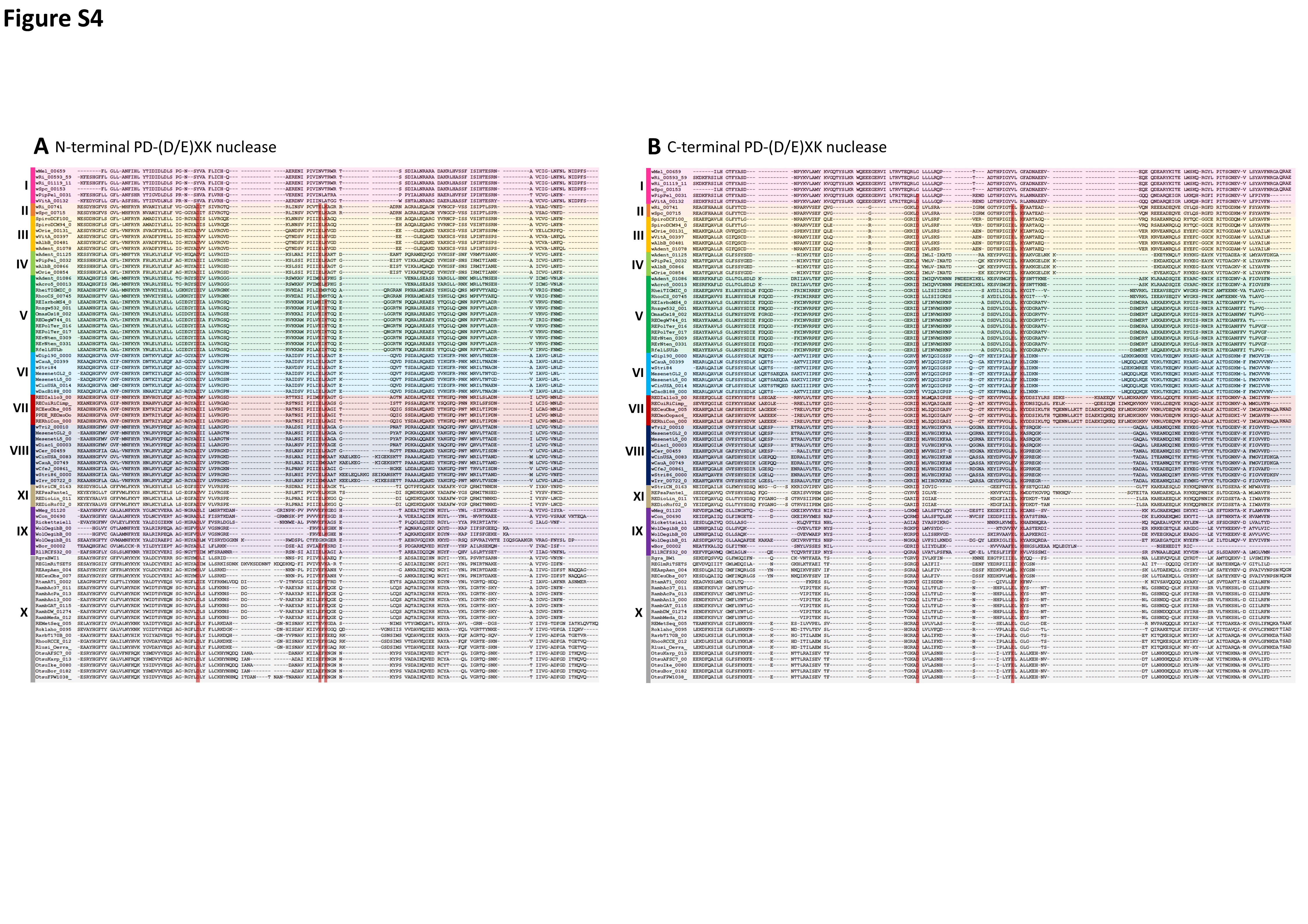

Supplement: S4 Fig — The three key catalytic residues ‘D–E–K’ are highlighted in red. The cif types I-X are indicated. (TIF) [file pgen.1011856.s011.TIF]

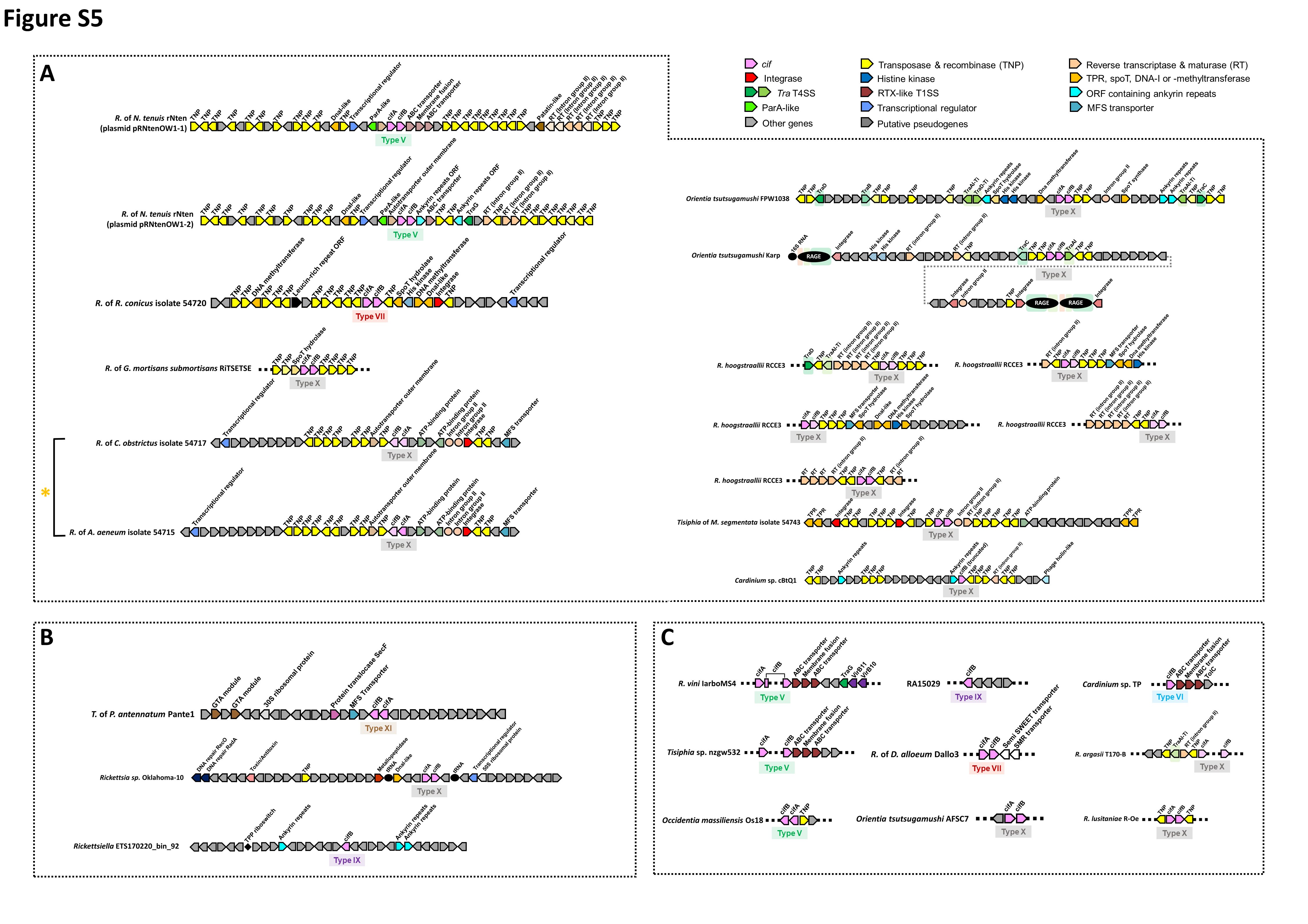

Supplement: S5 Fig — (A) Environment composed of small mobile genetic elements (SMGE) or RAGE-like. The yellow asterisk highlights homologous cif syntenic environments inherited through cladogenesis. (B) Environment devoid of mobile elements. (C) Environment interpretation limited by excessively small contigs. Gene lengths are not to scale. Cif type V, VII, XI and X are indicated below the bacterial strain names. (TIF) [file pgen.1011856.s012.tif]

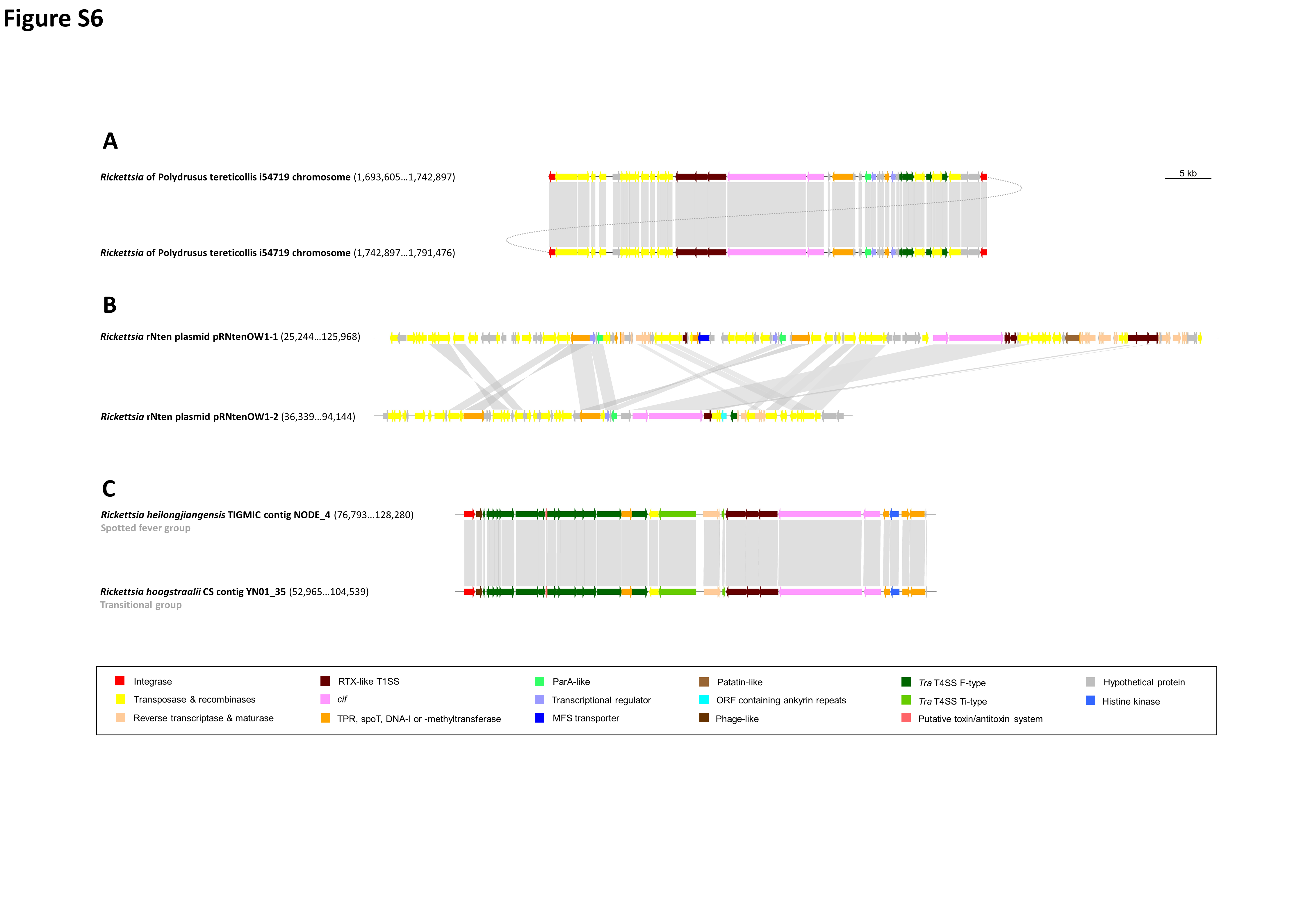

Supplement: S6 Fig — (A) Adjacent duplication of an intra-RAGE region flanked by two integrases. Gray dashed lines represent the continuation of the genome onto the next line for visualization purposes. (B) Plasmids densely covered with small mobile elements showing genomic rearrangements. (C) Identical RAGE structure identified on contigs of two unrelated Rickettsia strains. Their corresponding phylogenetic groups are indicated in grey. Position coordinates of the six represented genomic regions of the Rickettsia endosymbionts are indicated in parentheses. Gray lines connect orthologous genes. The gene delimitation lengths are relative to the indicated scale. (TIF) [file pgen.1011856.s013.tif]

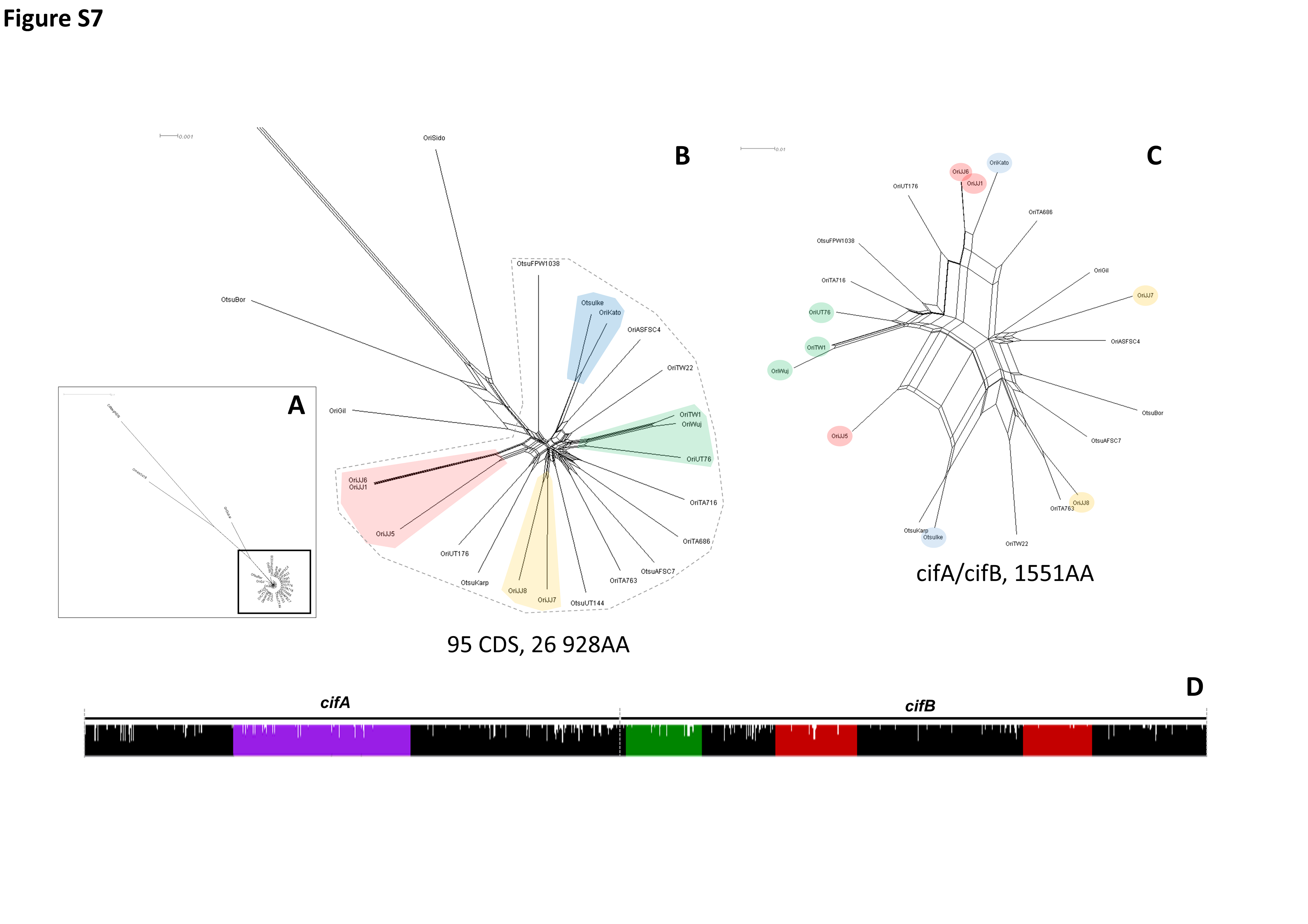

Supplement: S7 Fig — (A) Whole-genome phylogeny of the two Orientia species: O. tsutsugamushi and O. chuto (OriDubai). Occidentia massiliensis Os18 (OmasOs18) and Cd. Megaira MegNEIS296 (CdMegNEIS) were used as outgroups. The maximum-likelihood (ML) whole-genome phylogeny was constructed using 95 single-copy orthologs (SCO) (26,928 amino acids) extracted from the pangenome (CPREV+G4m substitution model). (B) Network analysis of the 95 concatenated SCOs using the Neighbor-Net method. Each edge (or set of parallel edges) represents a split in the dataset, with its length corresponding to the split’s weight. Clades supported by bootstrap values >89 are colored. (C) Network analysis of the cif pair from O. tsutsugamushi concatenated from 1,551 amino acids. The corresponding clade colors of strains carrying the cif pairs are represented. (D) Alignment of the 1,551 amino acids of the cifA-cifB pair. Protein domain locations are highlighted, corresponding to the colors used in Fig 2 (purple: RNA-binding-like; green: AAA-ATPase-like; red: PD-(D/E)XK nuclease). Residue conservation along the cif pair is shown by the height of each peak: solid peaks indicate high conservation, while fragmented peaks indicate low conservation at these positions. (TIF) [file pgen.1011856.s014.tif]

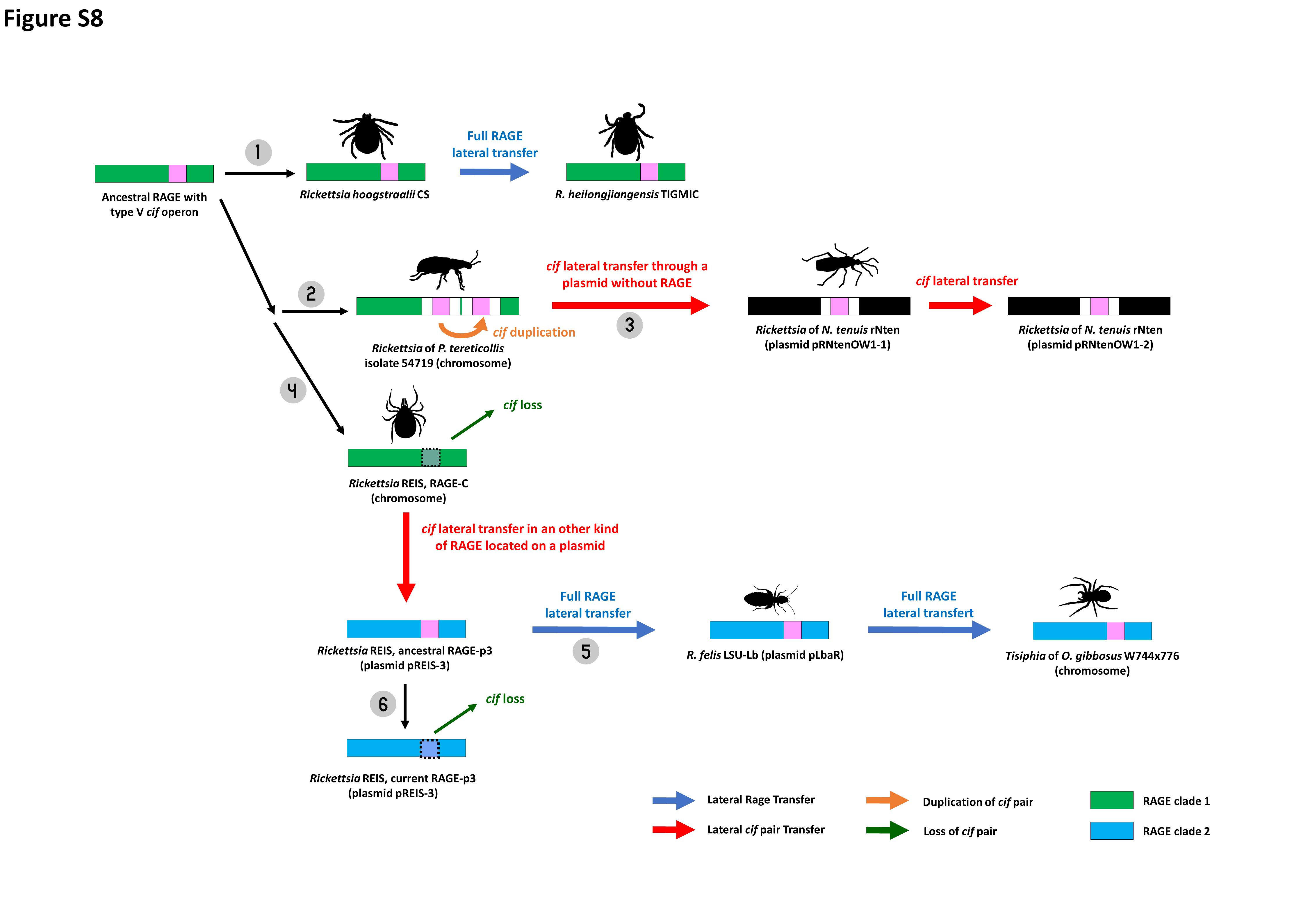

Supplement: S8 Fig — This scenario outlines how type V cif genes may have spread within Rickettsiaceae through an ancestral cif pair in a clade 1 RAGE module. (1) Horizontal transfer of a RAGE drove cif pair exchange between two unrelated Rickettsia species, possibly during co-infection in a same host. (2) In Rickettsia infecting the beetle Polydrusus tereticollis, a chromosomal region containing cif was duplicated within a RAGE, inherited from the ancestral clade 1 RAGE. (3) This cif pair, or a close relative, transferred into a plasmid of rNten, a Rickettsia infecting Nesidiocoris tenuis, via small mobile genetic elements (SMGEs), then moved to a second plasmid. (4) A type V cif pair was likely present in the chromosomal RAGE-C of the Rickettsia endosymbiont of Ixodes scapularis (REIS) before pseudogenization, possibly due to acquiring the biotin operon (vitamin B synthesis [62]), shifting its role from reproductive manipulator to nutritional mutualist. Previously, the cif pair may have proliferated between genomic compartments and moved onto the pREIS-3 plasmid within a clade 2 RAGE (RAGE-p3). (5) This clade 2 RAGE and its cif pair, or a close relative, transferred into a plasmid of another Rickettsia species infecting booklice (Liposcelis bostrychophila, Rickettsia felis strain LSU-Lb), then integrated into the chromosome of a Tisiphia infecting the spider Oedothorax gibbosus (Oegibbosus-W744x776). (6) In REIS, the RAGE-p3 has lost its putative cif pair. Open-source images available are used under permissive licenses from Openclipart (https://openclipart.org/share) and Pexels (https://www.pexels.com/license/). (TIF) [file pgen.1011856.s015.TIF]

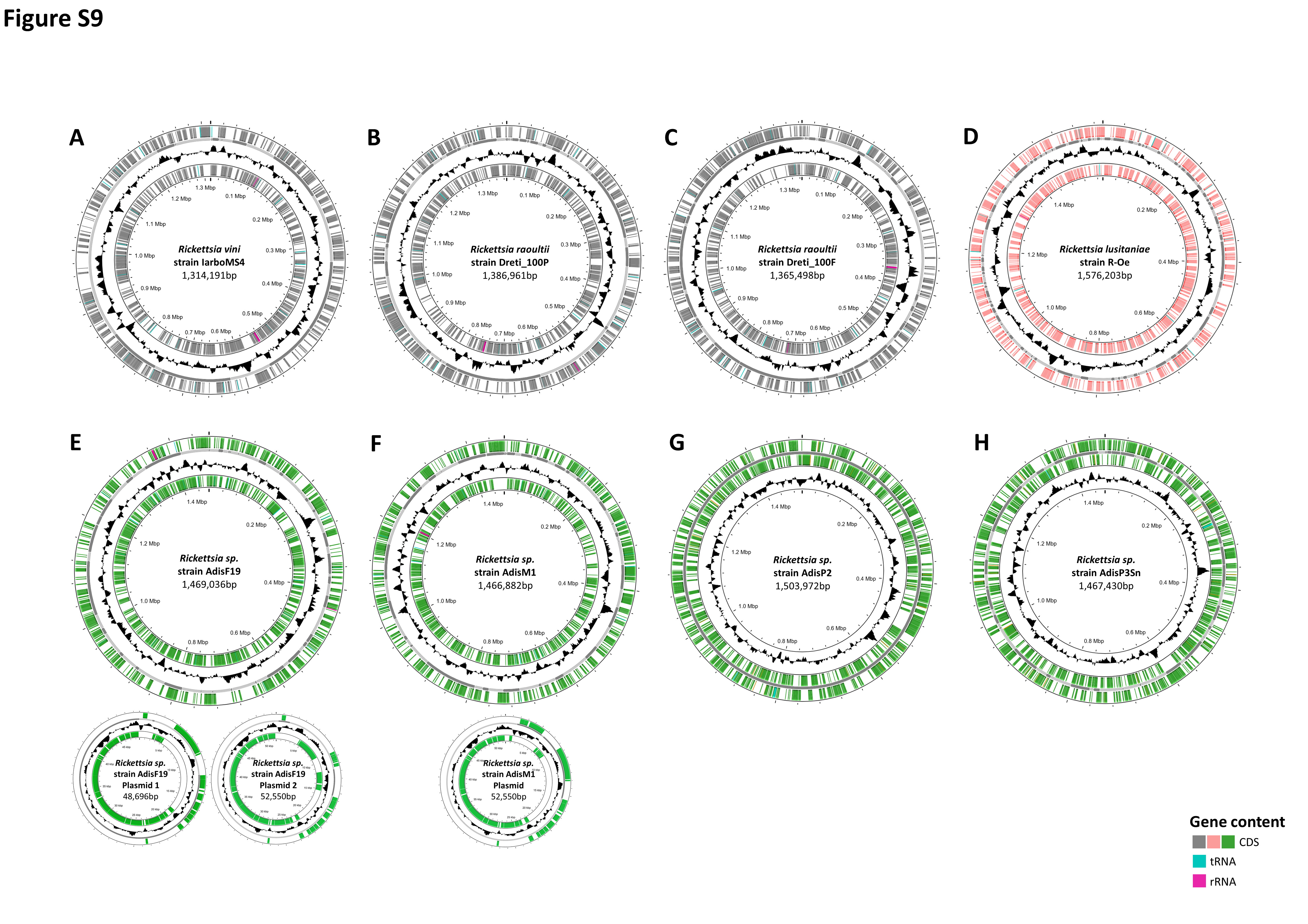

Supplement: S9 Fig — Circles on genome maps correspond to the following (from the edge to the middle): (1) forward strand genes; (2) contigs in dark and light gray; (3) GC contents; (4) reverse strand genes. In grey, genomes from the Spotted Fever Group (SFG); in pink, from the Transitional Group (TRG); in green, from the Belli Group (BEL). (TIF) [file pgen.1011856.s016.TIF]

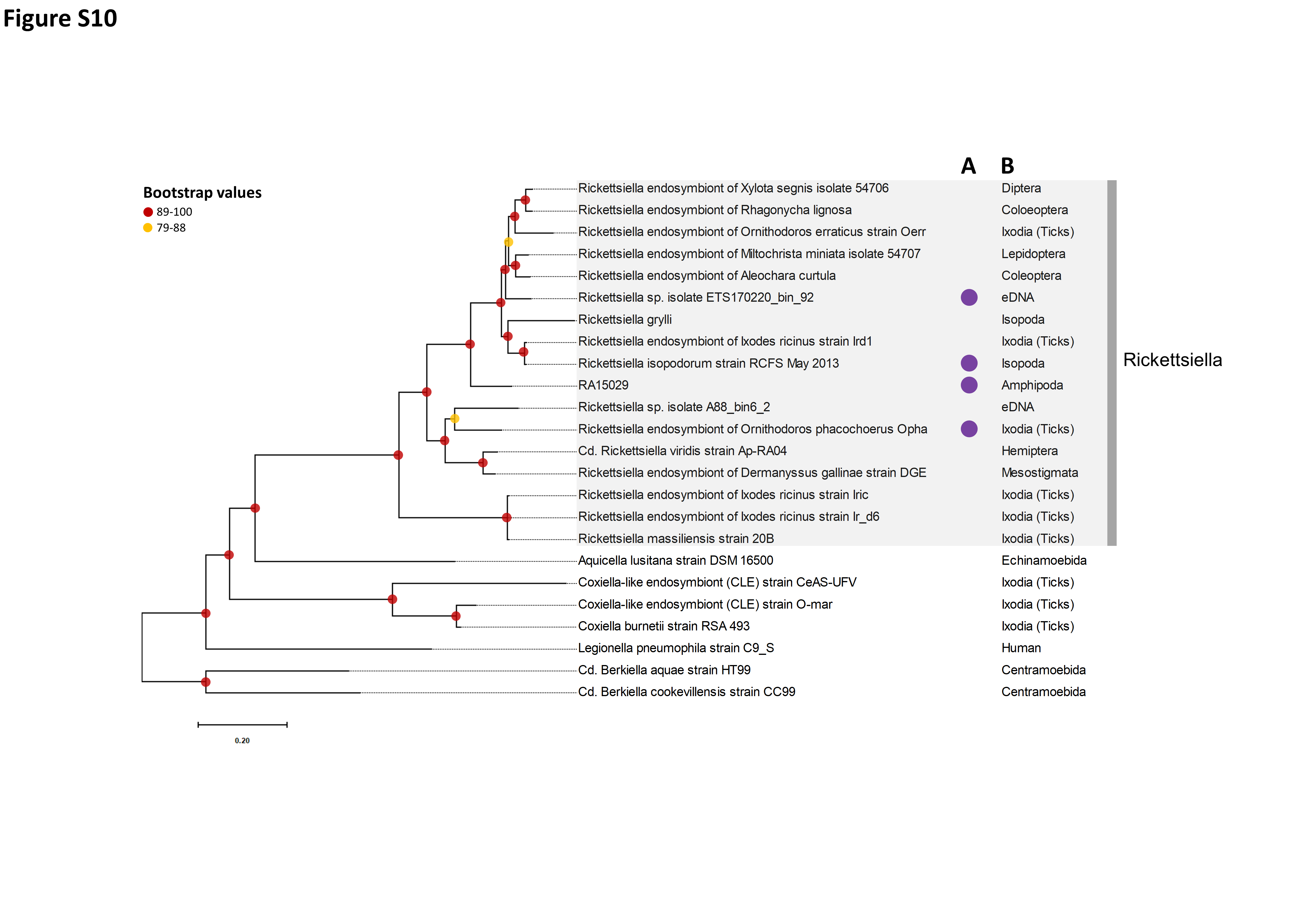

Supplement: S10 Fig — Genomes of Aquicella, Coxiella, Legionella, and Berkiella were used as outgroups. Bootstrap values were estimated from 1,000 replicates. (A) Presence of cif genes, with colors referring to cif type IX. (B) Host order from which the genome was isolated. (TIF) [file pgen.1011856.s017.TIF]
